# A Celastrol–Platinum(IV) Nanoprodrug Couples Ferroptosis With Caspase‐Independent Pyroptosis to Enhance Anti‐PD‐1 Therapy in Colorectal Cancer

**DOI:** 10.1002/advs.78681

**Published:** 2026-11-09

**Authors:** Huirong Li, Wenxiang Feng, Le Xin, Wu Zhong, Zhuhuai Peng, Zhihong Ke, Wenbin Zhou, Yutong Wu, Ying Chen, Hongwei Wang, Jidong Liu, Jinjun Ye, Weihong Guo

**Affiliations:** ^1^ Department of General Surgery Shenzhen Clinical College (Longgang Central Hospital of Shenzhen) Guangzhou University of Chinese Medicine Shenzhen China; ^2^ Department of Gastroenterological Surgery Ganzhou Hospital‐Nanfang Hospital Southern Medical University (Ganzhou People's Hospital) Ganzhou China

**Keywords:** cancer research, cd8, calreticulin, chemistry, hmgb1, immunotherapy, immunogenic cell death, programmed cell death, pyroptosis, tumor progression

## Abstract

Colorectal cancer (CRC) often responds incompletely to immunotherapy because tumor cells display limited immunogenicity and are embedded in an immunosuppressive microenvironment. Here, we engineered CPAN, a hyaluronic acid‐modified celastrol‐platinum(IV) nanoprodrug, to sensitize tumors to programmed cell death protein 1 (PD‐1) blockade by linking ferroptosis with caspase‐independent pyroptosis. Following tumor‐oriented delivery, CPAN perturbed redox balance through peroxiredoxin 1 (PRDX1) inhibition, glutathione (GSH) depletion, glutathione peroxidase 4 (GPX4) reduction, and heme oxygenase 1 (HO‐1) upregulation, which together promoted ferrous iron (Fe^2^^+^)‐associated lipid peroxidation. The platinum(IV) moiety concurrently activated poly(ADP‐ribose) polymerase (PARP)/PARylation signaling. These lipid reactive oxygen species (ROS)‐ and PARylation‐dependent events drove oligomerization of full‐length gasdermin E (GSDME), producing caspase‐independent pyroptotic death. The resulting immunogenic cell death (ICD) was evidenced by adenosine triphosphate (ATP) release, calreticulin (CRT) exposure, high mobility group box 1 (HMGB1) translocation, and increased dendritic cell (DC) maturation. In CT26 tumor‐bearing mice, CPAN restrained tumor progression, promoted CD8^+^ T‐cell infiltration, decreased the regulatory T‐cell (Treg) fraction, and enriched M1 macrophages. Anti‐PD‐1 treatment further amplified the antitumor effect of CPAN. These results highlight a promising nanoprodrug‐based strategy for improving CRC immunotherapy by coordinated activation of ferroptosis and caspase‐independent pyroptosis.

## Graphical abstract

CPAN integrates celastrol‐driven oxidative stress with Pt(IV)‐induced DNA damage to couple ferroptosis with caspase‐independent pyroptosis. Celastrol disrupts the GSH/GPX4 axis and promotes lipid peroxidation, while Pt(IV)‐associated PARP/PARylation signaling facilitates full‐length GSDME oligomerization. The resulting immunogenic cell death enhances CD8+ T‐cell responses and sensitizes colorectal tumors to anti‐PD‐1 therapy.

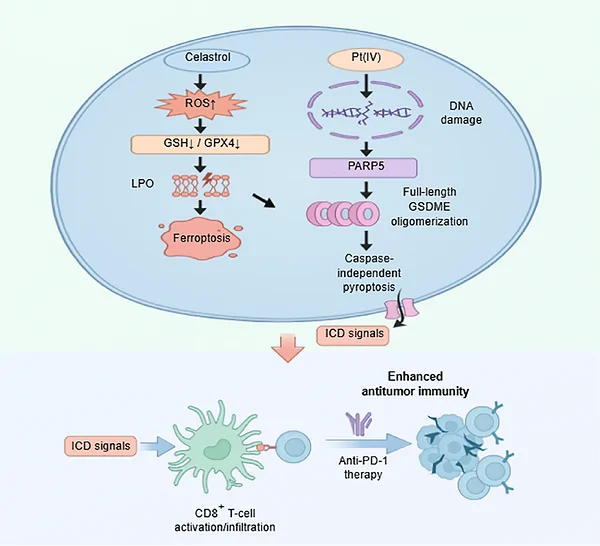

## 1 Introduction

Colorectal cancer (CRC) is one of the most common gastrointestinal malignancies worldwide, and patients with advanced, recurrent, or metastatic disease still have limited survival benefits [1]. In recent years, immune checkpoint blockade, especially programmed cell death protein 1 (PD‐1)/programmed death ligand 1 (PD‐L1) blockade, has changed the treatment landscape for a subset of patients with CRC [2, 3, 4]. This clinical benefit, however, is mainly observed in deficient mismatch repair/microsatellite instability‐high (dMMR/MSI‐H) tumors, whereas patients with the more prevalent proficient mismatch repair/microsatellite‐stable (pMMR/MSS) subtype generally derive limited benefit from single‐agent immune checkpoint blockade [5, 6, 7]. Limited tumor‐cell immunogenicity, inefficient antigen presentation, a strongly immunosuppressive microenvironment, and restricted effector T‐cell priming and infiltration all contribute to this resistance [8, 9, 10]. Therefore, inducing more immunogenic tumor‐cell death and converting immune‐low CRC into an immune‐activated state are important strategies for improving CRC immunotherapy [11].

Ferroptosis is an iron‐dependent regulated cell‐death process governed by lipid peroxide accumulation. Its key features include disruption of intracellular redox homeostasis, impairment of the glutathione/glutathione peroxidase 4 (GSH/GPX4) antioxidant defense system, ferrous iron (Fe^2^^+^) accumulation, and sustained amplification of membrane lipid peroxides [12, 13]. Recent studies suggest that ferroptosis can directly suppress tumor‐cell survival and remodel the tumor immune microenvironment by promoting lipid peroxidation products, damage‐associated molecular pattern (DAMP) release, and antigen presentation [14, 15, 16]. Thus, ferroptosis induction is considered a promising approach for increasing the immunogenicity of CRC cell death and improving immunotherapy response [17]. However, lipid peroxidation damage alone does not necessarily generate efficient membrane‐rupturing immunogenic death. A key challenge is how to couple ferroptosis‐associated oxidative lipid stress to stronger DAMP release and immune activation.

Pyroptosis is a programmed cell‐death modality marked by membrane pore formation, cellular swelling, membrane rupture, and release of inflammatory mediators [18, 19]. Compared with classical apoptosis, pyroptosis is often accompanied by tumor‐associated antigen release, DAMP exposure, and immune‐cell recruitment [20, 21]. It therefore has strong potential to turn immunologically cold tumors into hot tumors that respond more readily to immunotherapy. Classical pyroptosis usually depends on proteolytic cleavage of gasdermins by caspase‐3/1 or related proteases, which releases pore‐forming N‐terminal fragments and ruptures the plasma membrane [19, 22, 23]. In CRC cells, however, caspase‐dependent death execution may be compromised by reduced caspase expression or impaired activation, rendering caspase‐mediated gasdermin cleavage variable across different tumor contexts [19, 24, 25]. Even when oxidative stress causes cellular injury, tumor‐cell death may remain a poorly immunogenic damage or apoptotic state if it does not efficiently activate caspases and gasdermin pore formation. Exploring alternative strategies that bypass caspase cleavage and directly drive gasdermin membrane localization and pore formation may therefore improve the immunogenicity of CRC cell death and its response to immunotherapy. Notably, recent work has shown that gasdermin E (GSDME) can execute pyroptosis as a full‐length protein without proteolytic cleavage. Severe DNA damage activates nuclear poly(ADP‐ribose) polymerase 1 (PARP1) and induces abundant poly(ADP‐ribose) (PAR) polymer formation. PAR can then be released from the nucleus into the cytoplasm and promote GSDME PARylation. PARylation alters the conformation of full‐length GSDME and relieves its autoinhibited state, whereas lipid ROS‐mediated oxidative modification further promotes oxidative oligomerization, membrane localization, and pore formation by PARylated GSDME [26]. This process ultimately induces cleavage‐independent GSDME‐mediated pyroptosis. Accordingly, simultaneous amplification of lipid peroxidation and DNA damage in tumor cells may coordinate ferroptosis with caspase‐independent pyroptosis and enhance the immunogenicity of tumor‐cell death.

Celastrol is a natural product with multi‐target antitumor activity. It can induce reactive oxygen species (ROS) accumulation, disrupt redox homeostasis, and increase lipid peroxidation stress, making it suitable for amplifying ferroptosis‐associated stress [27, 28]. Platinum drugs, by contrast, induce DNA damage through DNA adduct formation and are classical chemotherapeutic agents [29]. Compared with platinum(II) (Pt(II)) drugs, platinum(IV) (Pt(IV)) prodrugs have greater structural stability and lower nonspecific reactivity, and their axial ligands can be modified to integrate functional molecules and construct nanoprodrugs [30, 31]. Integrating celastrol and Pt(IV) into a single prodrug system may therefore amplify lipid peroxidation stress and DNA damage at the same time. Molecular co‐delivery may also strengthen their intracellular synergy in tumor cells.

Based on this rationale, we constructed hyaluronic acid (HA)‐modified celastrol‐platinum(IV) amphiphilic prodrug self‐assembled nanoparticles (CPAN). HA has good biocompatibility and water solubility and can enhance CRC‐cell uptake and tumor accumulation through binding to the CD44 receptor. CPAN improves drug utilization through HA‐mediated targeted delivery. In tumor cells, it cooperatively amplifies ROS accumulation, lipid peroxidation, and DNA damage, thereby inducing ferroptosis and caspase‐independent GSDME‐associated pyroptotic membrane rupture. This dual‐regulated cell‐death process further increases calreticulin (CRT) exposure and high mobility group box 1 (HMGB1) and adenosine triphosphate (ATP) release, promotes dendritic cell maturation, and strengthens T‐cell‐mediated antitumor immunity. As a result, CPAN improves CRC responsiveness to PD‐1 blockade. This study provides a nanoprodrug strategy for enhancing CRC immunotherapy by coordinated induction of ferroptosis and caspase‐independent pyroptosis (Scheme 1).

**SCHEME 1:**
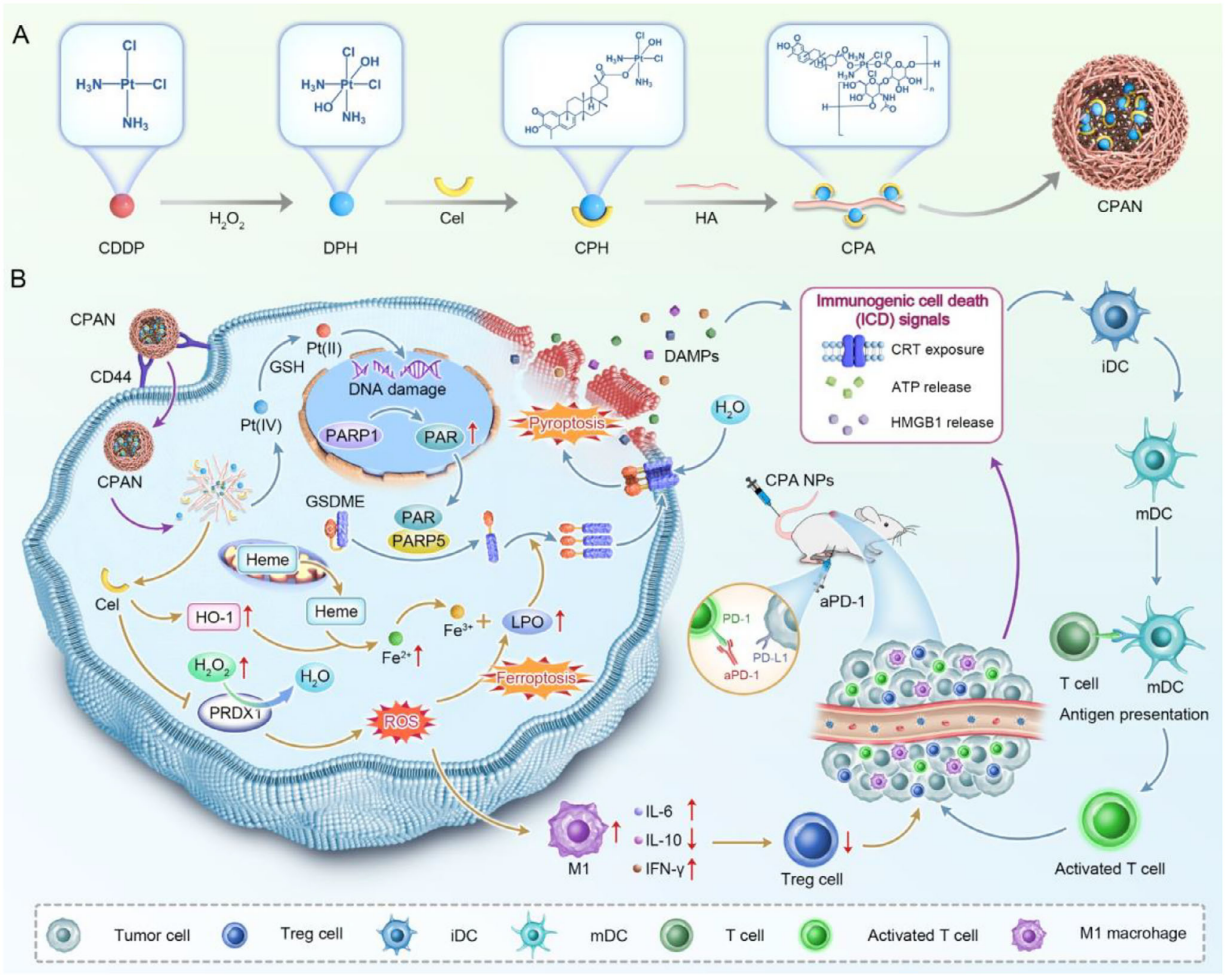
Schematic illustration of CPAN synthesis and its induction of ferroptosis and caspase‐independent, GSDME‐mediated pyroptosis to remodel the tumor immune microenvironment and enhance anti‐PD‐1 therapy in CRC.

## 2 Results and Discussion

### 2.1 Synthesis and Characterization of CPAN

Dihydroxyplatinum(IV) (DPH) was employed as the Pt(IV) precursor for construction of the nanoprodrug. Celastrol (Cel) and HA were incorporated through esterification to generate an amphiphilic HA‐conjugated Cel–Pt(IV) prodrug, which spontaneously assembled in aqueous media to form CPAN (Scheme 1A). The morphology of CPAN was first examined by transmission electron microscopy (TEM). As shown in Figure 1A, CPAN formed dispersed, near‐spherical nanoparticles with clear particle boundaries. Additional low‐magnification TEM and scanning electron microscopy (SEM) images provided further morphological characterization of CPAN (Figure S1). TEM‐based analysis indicated a dry‐state particle diameter of approximately 80 nm. Elemental mapping showed broadly overlapping distributions of C, N, O, and Pt within the particles, with the Pt signal colocalized with the nanoparticle profile, confirming incorporation of the Pt‐containing component (Figure 1B). Dynamic light scattering (DLS) analysis showed a larger Z‐average hydrodynamic diameter of approximately 135 nm with a PDI of 0.271 in aqueous dispersion (Figure 1C), consistent with the hydrated state of CPAN in solution. UV–vis spectroscopy, Fourier‐transform infrared (FTIR) spectroscopy, and proton nuclear magnetic resonance (^1^H NMR) spectroscopy analyses were then used to verify the successful synthesis of the HA‐conjugated CPH prodrug (CPA). As shown in Figure 1D, oxidation of CDDP to DPH shifted the absorption peak at 210–234 nm and altered the peak profile near 300 nm, suggesting a change in the Pt coordination environment. After celastrol introduction, celastrol–DPH conjugate (CPH) retained the characteristic absorption peaks of celastrol at 245 and 424 nm, while the Pt‐related absorption peaks shifted further. In CPA, the absorption peak at 290 nm was markedly weakened or disappeared, indicating that HA modification further altered the spectral features of the Pt(IV) prodrug (Figure 1E). FTIR spectra showed that the newly appearing O─H peak at 3511 cm^−^^1^ and the Pt─O vibration peaks at 557/452 cm^−^^1^ in DPH supported successful DPH synthesis. In CPH, the O─H peak shifted from 3511 to 3422 cm^−^^1^ and the carboxyl C═O peak of celastrol shifted from 1706 to 1693 cm^−^^1^, suggesting esterification between celastrol and DPH (Figure 1F). After HA grafting, CPA showed an ester‐bond C═O characteristic peak near 1739 cm^−^^1^ and retained absorption peaks associated with the HA sugar ring and CPH, supporting formation of the CPA conjugate (Figure 1G). In the ^1^H NMR spectrum, CPA showed proton signals corresponding to Pt ammine ligands, celastrol methyl/methylene groups, and HA acetyl groups (Figure 1H). The HA substitution degree calculated from ^1^H integration was 33.3%, further confirming that Pt(IV), celastrol, and HA were successfully integrated into the same prodrug structure.

**FIGURE 1:**
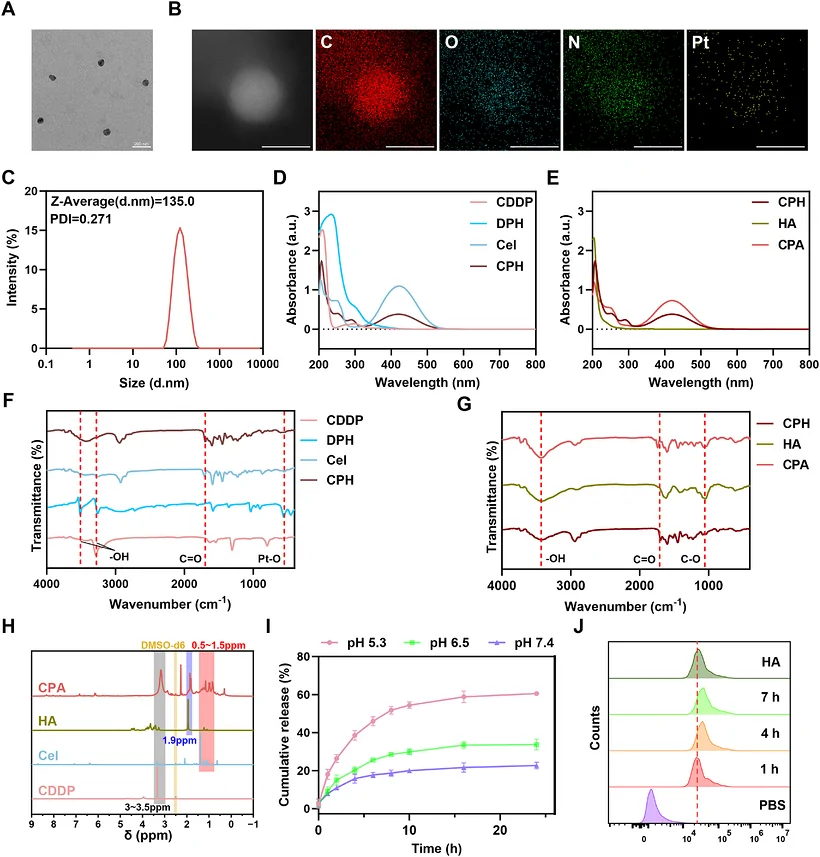
Construction, physicochemical properties, and characterization of CPAN. (A) TEM morphology of CPAN (scale bar, 200 nm). (B) Elemental distributions of C, O, N, and Pt within CPAN determined by elemental mapping (scale bar, 100 nm). (C) Hydrodynamic‐size profile obtained by DLS. (D,E) UV–vis spectra of CDDP, DPH, Cel, CPH, HA, and CPA. (F,G) FTIR spectra of the indicated precursors and conjugates. (H) ^1^H NMR spectra of CDDP, Cel, HA, and CPA. (I) Time‐dependent cumulative Cel release from CPAN at pH 5.3, 6.5, or 7.4. (J) Flow‐cytometric assessment of rhodamine B‐labeled CPAN internalization by CT26 cells at different incubation times and following competitive blockade with free HA.

We further evaluated the stability of CPAN under different dispersion and storage conditions. As shown in Figure S2A–D, the Z‐average diameter and PDI remained largely unchanged in deionized water, phosphate‐buffered saline (PBS), and PBS containing 5% bovine serum albumin (BSA) at 37°C, as well as during storage at 4°C or 25°C for 7 d. Lyophilized CPAN also showed no significant changes in particle size or PDI after storage at 25°C for 7 d and subsequent reconstitution (Figure S2E,F), while no visible precipitation or phase separation was observed in the aqueous dispersions (Figure S2G). These results indicate good short‐term colloidal and storage stability of CPAN. We next examined the in vitro release behavior of CPAN. As shown in Figure 1I, cumulative drug release reached 60.5% and 33.7% within 24 h at acidic pH values of 5.3 and 6.5, respectively, indicating rapid and substantial drug release under mildly acidic conditions. By contrast, cumulative release under physiological pH (pH 7.4) was much lower over the same period, reaching only 22.6%. This difference in release kinetics highlights the pH responsiveness of CPAN nanoparticles, which release drug more rapidly at low pH. CPAN is therefore expected to release its payload effectively in the tumor microenvironment (pH 6.5) and in tumor‐cell lysosomes (pH 5.3), enabling functional therapeutic activity. Finally, we evaluated the cellular uptake of CPAN and the involvement of HA–CD44 recognition in this process. Western blotting and immunofluorescence staining confirmed CD44 expression in CT26 cells (Figure S3). As shown in Figure 1J and Figure S4, intracellular fluorescence from rhodamine B‐labeled CPAN increased with incubation time in CT26 cells. Pretreatment with free HA markedly reduced the fluorescence signal, suggesting the involvement of HA‐CD44 recognition in CPAN uptake.

Together, these results confirm the successful synthesis of CPAN. The nanoparticles have a defined nanostructure, good colloidal stability, efficient HA‐mediated cellular uptake, and microenvironment‐responsive release, providing a foundation for subsequent induction of tumor‐cell death and immune sensitization.

### 2.2 CPAN Induces Ferroptosis and Caspase‐Independent Pyroptosis in Tumor Cells and Exerts In Vitro Antitumor Activity

To evaluate the cytotoxicity of CPAN nanoparticles toward CT26 cells, we measured cell viability after treatment with different CPAN concentrations using the Cell Counting Kit‐8 (CCK‐8) assay. As shown in Figure 2A, CPAN significantly reduced CT26‐cell viability in a concentration‐dependent manner, with a half‐maximal inhibitory concentration (IC50) value of 1.081 µg mL^−^^1^. We then compared the cytotoxicity of different treatments at the same celastrol‐equivalent concentration. As shown in Figure 2B, CDDP alone only slightly reduced CT26‐cell viability, whereas celastrol and CPAN produced stronger inhibition at the same celastrol‐equivalent concentration; CPAN showed the strongest inhibitory effect. Calcein‐AM/PI live/dead fluorescence staining further confirmed the cell‐killing effect of CPAN. As shown in Figure 2D,E, the CPAN group contained a large number of PI‐positive dead cells, far exceeding the other control groups. These results indicate that CPAN induces stronger cell death than free celastrol, CDDP, or their simple combination (CP), suggesting that the nanoprodrug format improves effective drug action in tumor cells.

**FIGURE 2:**
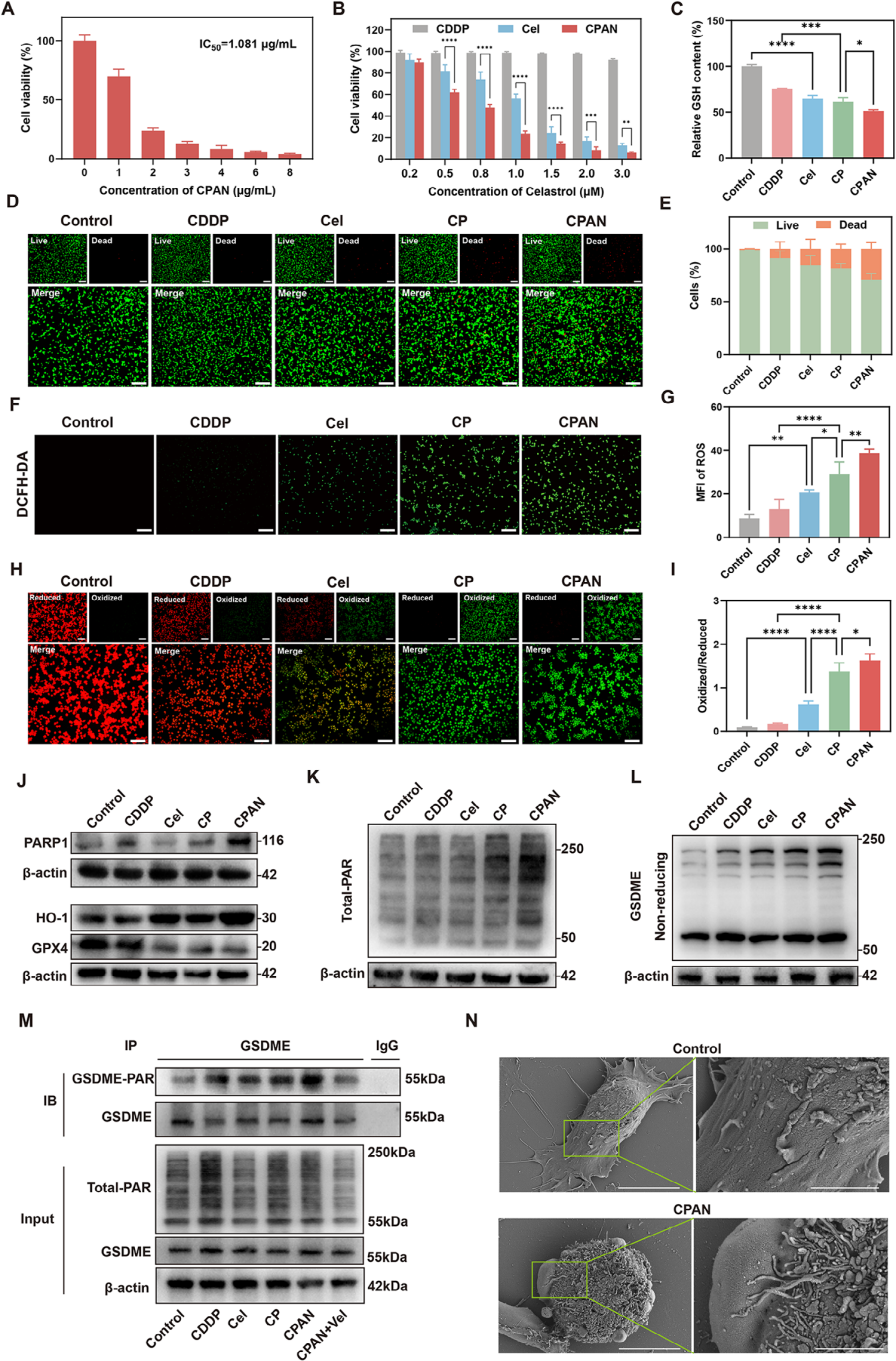
CPAN induces ferroptosis and caspase‐independent GSDME pyroptosis in CT26 cells in vitro. (A) Concentration‐dependent effect of CPAN on CT26 viability and determination of IC50 by CCK‐8 assay. (B) Cell viability following CDDP, Cel, or CPAN exposure at matched Cel‐equivalent concentrations. (C) Intracellular GSH levels after the indicated treatments. (D,E) Representative Calcein‐AM/PI images and corresponding live/dead‐cell quantification (scale bar, 200 µm). (F,G) DCFH‐DA fluorescence and quantitative MFI analysis of intracellular ROS (scale bar, 200 µm). (H,I) C11‐BODIPY 581/591 imaging and oxidized/reduced fluorescence ratios reflecting lipid oxidation (scale bar, 200 µm). (J) PARP1, HO‐1, and GPX4 expression evaluated by Western blotting. (K) Immunoblot detection of total PAR. (L) Detection of high‐molecular‐weight GSDME species under non‐reducing conditions. (M) Co‐IP assessment of GSDME‐associated PARylation. (N) SEM images illustrating CPAN‐induced morphological alterations in CT26 cells (scale bars, 10 µm, left; 3 µm, right). CP represents free CDDP plus Cel. Results are expressed as mean ± SD (*n* = 3) and were analyzed by one‐way ANOVA. ns, not significant; *p < 0.05, **p < 0.01, ***p < 0.001, ****p < 0.0001.

After confirming the marked cytotoxicity of CPAN, we investigated the underlying mechanism of cell death. Previous studies have shown that celastrol can interfere with peroxiredoxin 1 (PRDX1)‐mediated peroxide clearance and induce heme oxygenase 1 (HO‐1) upregulation, thereby amplifying intracellular oxidative stress and inducing ferroptosis [27, 32]. PRDX1 is an important intracellular H_2_O_2_‐scavenging enzyme, and inhibition of its activity can lead to ROS accumulation [33]. HO‐1, a heme‐metabolism‐related enzyme, may increase intracellular labile iron under sustained oxidative stress and further enhance lipid peroxidation [32, 34]. Glutathione peroxidase 4 (GPX4) is a key GSH‐dependent enzyme that removes lipid peroxides. Impairment of its expression or function causes continuous lipid peroxide accumulation and triggers ferroptosis [35]. We therefore first measured changes in GSH, ROS, Fe^2^^+^, lipid peroxidation, and GPX4/HO‐1 in CT26 cells after CPAN treatment. CPAN reduced intracellular GSH levels by 48.8%, whereas free CDDP and celastrol reduced GSH by 24.3% and 35.5%, respectively (Figure 2C). This result indicates that CPAN more strongly weakens the antioxidant defense system of tumor cells. To determine whether celastrol directly affects PRDX1‐mediated H_2_O_2_ clearance, we performed an in vitro enzyme activity assay using recombinant mouse PRDX1 (rmPRDX1). CDDP had no obvious effect on the H_2_O_2_‐scavenging ability of rmPRDX1, whereas celastrol markedly reduced rmPRDX1 activity and decreased its H_2_O_2_‐scavenging capacity to 42.3% of the control level (Figure S5). This result shows that the celastrol component of CPAN can directly weaken PRDX1‐mediated peroxide clearance, providing an important mechanism for CPAN‐induced intracellular ROS accumulation. We then detected intracellular ROS in CT26 cells using the DCFH‐DA probe. The CPAN group showed a strong increase in green fluorescence, with a mean fluorescence intensity approximately 4.4‐fold that of the control group (Figure 2F,G). These results indicate that CPAN releases celastrol to weaken both the GSH‐dependent antioxidant system and PRDX1‐mediated H_2_O_2_ clearance, promoting ROS accumulation and disrupting intracellular redox balance. Ferroptosis depends not only on ROS accumulation but also on sustained amplification of iron‐dependent lipid peroxidation [12]. We therefore examined whether CPAN induced Fe^2^^+^ accumulation and enhanced lipid peroxidation in CT26 cells. Fe^2^^+^ detection showed that intracellular Fe^2^^+^ increased markedly after CPAN treatment, reaching approximately 3.1‐fold the control level (Figure S6), suggesting that CPAN induced labile ferrous iron accumulation and provided an iron‐dependent basis for lipid peroxidation. We then used the BODIPY 581/591 C11 fluorescent probe to assess membrane lipid oxidative damage. CPAN markedly increased lipid peroxidation fluorescence in CT26 cells, and the signal was stronger than that induced by free celastrol, CDDP, or their combined treatment (Figure 2H,I), indicating effective amplification of lipid peroxidation injury. To further verify ferroptosis‐related changes, we measured GPX4 and HO‐1 expression by Western blotting. As shown in Figure 2J and Figure S7a,b, CPAN markedly reduced GPX4 expression and increased HO‐1 expression. We also performed a functional rescue experiment using the HO‐1 inhibitor zinc protoporphyrin IX (ZnPPIX). ZnPPIX pretreatment markedly weakened CPAN‐induced lipid peroxidation fluorescence (Figure S8), indicating that HO‐1 participates in CPAN‐mediated amplification of lipid peroxidation. A CCK‐8 rescue experiment using the ferroptosis inhibitor Liproxstatin‐1 further showed that Liproxstatin‐1 significantly inhibited CPAN‐induced cytotoxicity (Figure S9). Collectively, these results show that CPAN induces ferroptosis in CRC cells by inhibiting PRDX1‐mediated H_2_O_2_ clearance, weakening the GSH/GPX4 antioxidant defense, and promoting HO‐1/Fe^2^^+^‐associated lipid peroxidation.

Previous work has shown that lipid peroxidation and DNA damage‐associated PAR signaling can jointly trigger cleavage‐independent GSDME‐mediated pyroptosis [26]. Based on the design of CPAN, we examined whether it activated DNA damage‐associated signaling by measuring PARP1 and downstream PARylation levels. As shown in Figure 2J,K and Figure S7c,d, CPAN treatment markedly increased PARP1 expression and raised total PAR levels by approximately 126% compared with the blank control. We then used non‐reducing Western blotting to assess GSDME oligomerization and determine whether CPAN induced GSDME‐associated caspase‐independent pyroptosis. As shown in Figure 2L and Figure S7e,f, GSDME was mainly present as a monomer in control cells, whereas CPAN treatment produced clear high‐molecular‐weight GSDME oligomer bands. To further determine whether GSDME was associated with PARylation after CPAN treatment, GSDME was immunoprecipitated and then immunoblotted with an anti‐PAR antibody. The co‐immunoprecipitation (Co‐IP) assay showed that CPAN markedly increased the PAR signal in GSDME immunoprecipitates, whereas pretreatment with the PARP inhibitor Veliparib reduced this signal (Figure 2M and Figure S10). These results suggest that CPAN promotes GSDME‐associated PARylation in CT26 cells, accompanied by full‐length GSDME oligomerization. We also measured LDH levels in the tumor‐cell supernatants from different treatment groups. LDH release increased significantly after CPAN treatment (Figure S11). These results suggest that CPAN may promote GSDME oligomerization through PARP1/PARylation‐associated stress and thereby induce caspase‐independent pyroptosis. Light microscopy and scanning electron microscopy provided further morphological evidence. Under light microscopy, CPAN‐treated cells showed swelling and membrane blebbing‐like changes (Figure S12). Scanning electron microscopy showed that untreated CT26 cells had typical adherent spreading morphology, clear cell contours, relatively intact membranes, and obvious pseudopod extension. By contrast, CPAN‐treated CT26 cells showed marked rounding and swelling, roughened membrane surfaces, numerous membrane bleb‐like protrusions, and local membrane rupture, consistent with pyroptotic morphology (Figure 2N).

In summary, CPAN amplifies lipid peroxidation injury through celastrol‐mediated ROS accumulation and disruption of the GSH/GPX4 axis, thereby inducing ferroptosis. At the same time, Pt(IV)‐associated DNA damage stress activates PARP1/PARylation and promotes GSDME oligomerization, leading to caspase‐independent pyroptosis.

### 2.3 Inhibitor Rescue and Transcriptomic Analyses Reveal the Mechanism of CPAN‐Induced GSDME‐Associated Cell Death

To determine whether CPAN‐induced GSDME‐associated cell death depended on the classical caspase pathway, we performed rescue experiments using the pan‐caspase inhibitor Z‐VAD‐FMK. CCK‐8 assays showed that Z‐VAD‐FMK alone had no obvious effect on CT26‐cell viability. Compared with CPAN treatment alone, Z‐VAD‐FMK pretreatment also failed to significantly restore the CPAN‐induced decrease in cell viability. By contrast, the PARP5 inhibitor XAV‐939 alone showed no obvious cytotoxicity, but XAV‐939 pretreatment partially reversed the CPAN‐induced reduction in cell viability (Figure 3A). These results indicate that CPAN‐induced cell death is not mainly caspase‐dependent and that PARP5‐related signaling participates in this process. We then used non‐reducing Western blotting to examine the effects of different inhibitors on GSDME oligomerization. CPAN treatment markedly increased high‐molecular‐weight GSDME oligomers in CT26 cells. Pretreatment with the PARP inhibitor Veliparib or the lipid peroxidation inhibitor Liproxstatin‐1 reduced GSDME oligomerization, and the combined pretreatment produced a stronger inhibitory effect (Figure 3B and Figure S13). These findings support the conclusion that CPAN promotes caspase‐independent pyroptosis through the combined action of PARP/PARylation‐associated stress and lipid peroxidation accumulation.

**FIGURE 3:**
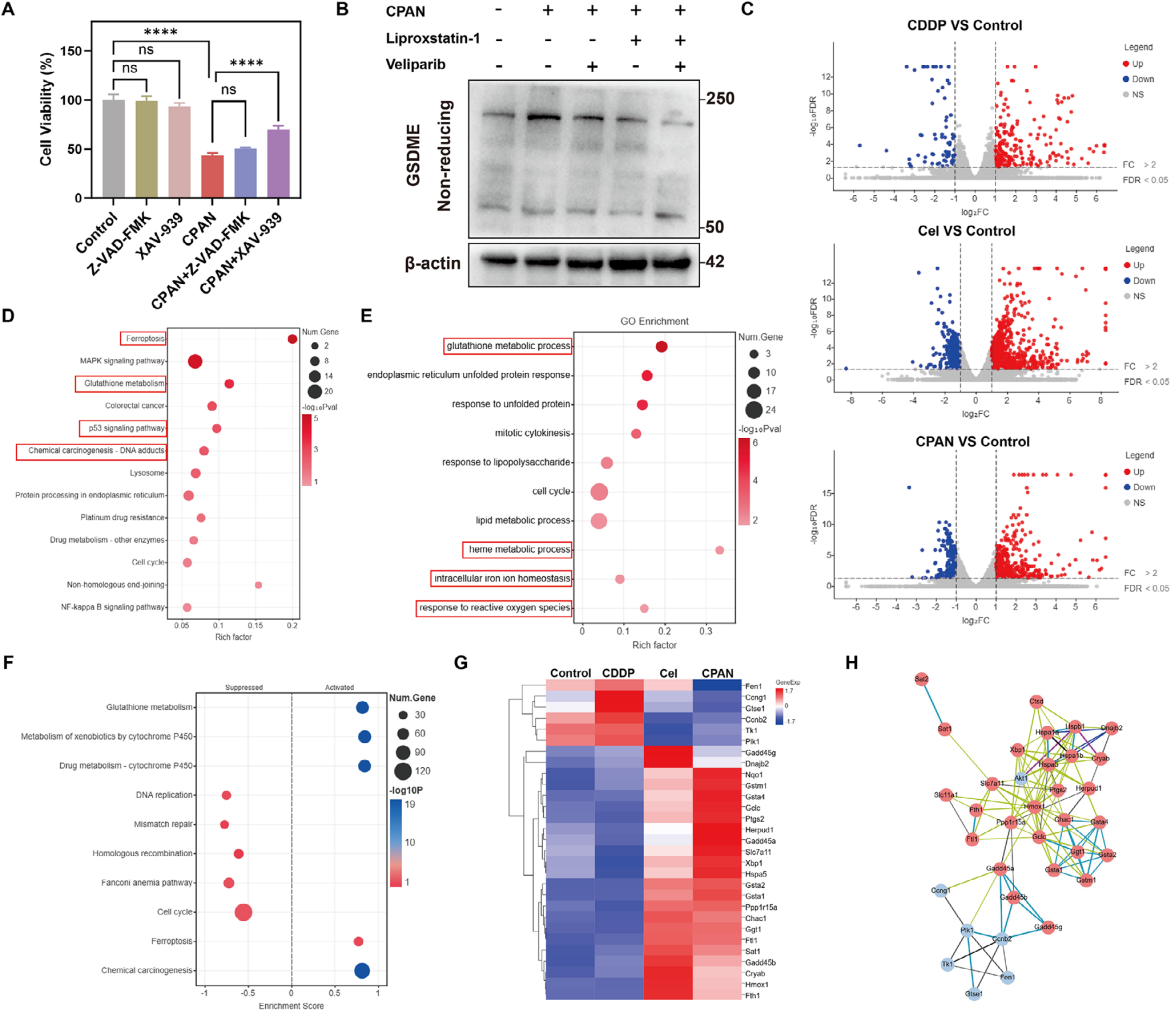
Inhibitor rescue and transcriptomic analyses reveal the molecular mechanism of CPAN‐induced GSDME‐associated cell death. (A) CT26 viability after CPAN treatment with or without Z‐VAD‐FMK or XAV‐939 pretreatment. (B) Non‐reducing immunoblot analysis of CPAN‐induced GSDME oligomers following pretreatment with Veliparib, Liproxstatin‐1, or their combination. (C) Volcano plots comparing transcriptional changes induced by CDDP, Cel, or CPAN relative to Control. (D) KEGG enrichment profile of CPAN‐responsive genes. (E) GO biological‐process enrichment of differentially expressed genes following CPAN exposure. (F) GSEA of treatment‐associated pathway alterations. (G) Heatmap of representative differentially expressed genes. (H) PPI network generated from selected CPAN‐responsive genes. Quantitative results are shown as mean ± SD (*n* = 3) and were analyzed using one‐way ANOVA. ns, not significant; ****p < 0.0001.

To further define the molecular basis of the cell‐death phenotype induced by CPAN at the transcriptional level, we performed RNA sequencing (RNA‐seq) analysis of CT26 cells treated with PBS, CDDP, celastrol, or CPAN. Differential expression analysis identified 293 differentially expressed genes after CDDP treatment compared with PBS, including 223 upregulated and 70 downregulated genes. Celastrol treatment produced 1,194 differentially expressed genes, including 780 upregulated and 414 downregulated genes. CPAN treatment produced 603 differentially expressed genes, including 427 upregulated and 176 downregulated genes (Figure 3C). At the pathway level, Kyoto Encyclopedia of Genes and Genomes (KEGG) enrichment analysis showed that differentially expressed genes after CPAN treatment were mainly enriched in ferroptosis, glutathione metabolism, chemical carcinogenesis‐DNA adducts, the p53 signaling pathway, and non‐homologous end joining (Figure 3D). This suggests activation of ferroptosis‐ and DNA damage‐related stress programs in CT26 cells after CPAN treatment. Gene set enrichment analysis (GSEA) further clarified the direction of these pathway changes. Glutathione metabolism, ferroptosis, chemical carcinogenesis/DNA adduct‐related pathways, and drug metabolism‐related pathways tended to be activated after CPAN treatment. By contrast, pathways related to DNA replication, cell cycle, mismatch repair, homologous recombination, and Fanconi anemia were suppressed (Figure 3F). These changes are consistent with the rescue experiments showing that PARP/PARylation and lipid peroxidation jointly participate in GSDME oligomerization. Gene Ontology (GO) enrichment analysis further supported this interpretation at the biological‐process level. Differentially expressed genes in the CPAN group were mainly enriched in glutathione metabolic process, response to reactive oxygen species, heme metabolic process, intracellular iron ion homeostasis, and lipid metabolic process (Figure 3E), indicating that CPAN systematically remodels redox homeostasis, heme metabolism, iron homeostasis, and lipid metabolism. These changes provide transcriptomic support for lipid ROS accumulation and ferroptosis‐associated lipid peroxidation.

To identify key transcriptional changes induced by CPAN, we analyzed representative genes related to oxidative stress, ferroptosis, and DNA damage. Upregulation of *Hmox1* was consistent with the increased HO‐1 protein expression described above, further supporting the involvement of HO‐1/Fe^2^^+^‐associated lipid peroxidation in CPAN‐induced ferroptosis. At the same time, DNA damage‐response genes such as *Gadd45a*, *Gadd45b*, and *Gadd45g* were upregulated, whereas DNA replication‐ and proliferation‐related genes such as *Plk1*, *Ccnb2*, *Tk1*, *Fen1*, and *Gtse1* were downregulated. These changes suggest that CPAN induces DNA damage‐associated stress together with suppression of proliferative programs (Figure 3G). Protein relationships within the related pathways were visualized using a protein–protein interaction (PPI) network (Figure 3H).

### 2.4 CPAN Induces Immunogenic Cell Death (ICD) in Colorectal Cancer Cells

ICD is a form of cell death with immune‐activating capacity. ICD is characterized by the release of DAMPs, including calreticulin (CRT) exposure, adenosine triphosphate (ATP) efflux, and high mobility group box 1 (HMGB1) release [36]. These released DAMPs can be recognized and taken up by dendritic cells (DCs), promoting DC maturation, T‐cell activation, and ultimately a strong antitumor immune response [37]. Our preceding results showed that CPAN efficiently induced ferroptosis and caspase‐independent pyroptosis in CT26 cells in vitro. Because ferroptosis and pyroptosis are immunogenic forms of cell death, both can induce ICD (Figure 4A). We therefore evaluated whether CPAN‐induced tumor‐cell death could trigger ICD and promote bone marrow‐derived dendritic cells (BMDCs) maturation.

**FIGURE 4:**
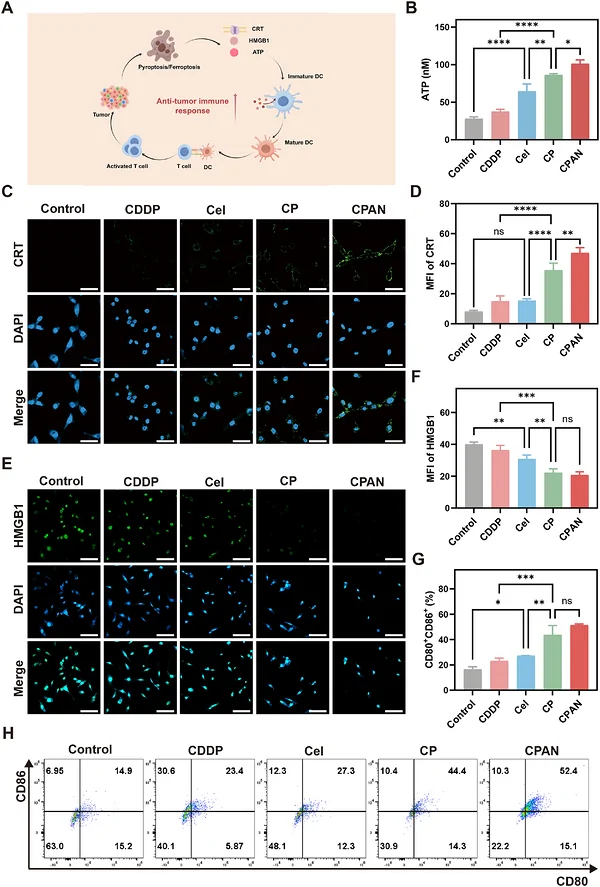
CPAN‐triggered immunogenic cell death in CT26 cells and BMDC maturation. (A) Schematic depiction of DAMP release, DC maturation, and antitumor immune activation after CPAN‐induced ferroptosis/pyroptosis. (B) Extracellular ATP measured in conditioned media from treated CT26 cells. (C,D) Immunofluorescence visualization and MFI quantification of CRT exposure (scale bar, 50 µm). (E,F) HMGB1 redistribution from the nucleus and corresponding fluorescence quantification (scale bar, 100 µm). (G,H) Representative flow‐cytometry profiles and frequencies of CD80^+^CD86^+^ mature BMDCs after exposure to CT26‐conditioned media. Quantitative data are mean ± SD (*n* = 3) and were evaluated by one‐way ANOVA. ns, not significant; *p < 0.05, **p < 0.01, ***p < 0.001, ****p < 0.0001. The schematic illustration in panel A was prepared using Figdraw.

We first measured ATP levels in the supernatant of treated CT26 cells. CPAN treatment markedly increased extracellular ATP to 101.4 nm, which was significantly higher than in the PBS, CDDP, and free celastrol groups (Figure 4B). We then used immunofluorescence to examine CRT exposure on the CT26‐cell membrane. CRT signals in control cells were weak and mainly intracellular. After CPAN treatment, CRT fluorescence around the cell membrane increased markedly, and quantitative analysis showed an approximately 4.7‐fold increase in fluorescence intensity relative to the control group (Figure 4C,D). HMGB1 release is another important marker of ICD. Immunofluorescence showed that HMGB1 was mainly localized in the nucleus in control cells. After CPAN treatment, HMGB1 translocated from the nucleus to the cytoplasm and was released, with a clear decrease in nuclear signal (Figure 4E,F). Together, ATP release, CRT exposure, and HMGB1 translocation/release indicate that CPAN‐induced ferroptosis and pyroptosis effectively trigger ICD in CT26 cells.

To determine whether these ICD signals promoted activation of antigen‐presenting cells, conditioned medium from CPAN‐treated CT26 cells was cocultured with BMDCs, and expression of the DC maturation markers CD80 and CD86 was measured by flow cytometry. The CPAN group induced the highest maturation rate (51.4%), significantly above that of the Control group (16.4%) (Figure 4G,H). Overall, these findings indicate that CPAN‐induced cell death enhances ICD and thereby elicits a strong immune response.

### 2.5 In Vivo Tumor Targeting, Safety, and Antitumor Efficacy of CPAN

In vitro, CPAN showed efficient tumor‐cell uptake and induced ferroptosis, caspase‐independent pyroptosis, and ICD. To further assess its potential for in vivo application, we first evaluated the biodistribution and tumor accumulation of CPAN. Small‐animal in vivo imaging showed that CPAN gradually accumulated at the tumor site after administration, with a strong fluorescence signal at 16 h. Ex vivo organ imaging further showed that CPAN retained high accumulation in tumor tissue, with some distribution in the liver and kidney (Figure S14). These findings indicate favorable tumor delivery of CPAN, while its distribution in the liver and kidney may be associated with systemic disposition and clearance. The long‐term tissue retention and clearance of CPAN, however, require further investigation. We then evaluated the in vivo safety of CPAN. BALB/c mice received tail‐vein injections of saline or CPAN every 3 days for a total of four injections. After 14 d, peripheral blood and major organs were collected for analysis. Serum biochemical results showed no obvious abnormalities in liver and kidney function indicators, including alanine aminotransferase (ALT), aspartate aminotransferase (AST), creatinine (CREA), and UREA, after CPAN treatment (Figure S15a). Hematoxylin and eosin (H&E) staining of major organs (heart, liver, spleen, lung, and kidney) showed essentially intact tissue structures in the CPAN group, with no obvious pathological changes (Figure S15b). Although these results support the short‐term biosafety of CPAN, its long‐term hematological toxicity, systemic toxicity, and immunotoxicity require further evaluation.

After confirming the biosafety and tumor‐targeting effect of CPAN, we evaluated the antitumor efficacy of different treatments (PBS, CDDP, Cel, CP, and CPAN) in BALB/c mice bearing CT26 tumors. As shown in Figure 5A, mice received four consecutive tail‐vein injections of different drugs. Cel and CDDP were administered at 3 and 2.0 mg kg^−^^1^, respectively. CP and CPAN were administered at the same Cel‐equivalent dose. CPAN significantly inhibited tumor growth compared with Control, CDDP, and Cel groups, and no obvious body‐weight loss was observed during treatment (Figure 5B–I). Tumor tissues from each group were then collected for hematoxylin and eosin (**H&E**) staining and Ki‐67 immunohistochemical staining. In the Control group, tumor cells showed mitotic nuclei, obvious atypia, and many Ki‐67‐positive cells, indicating active tumor‐cell proliferation. In the CPAN‐treated group, obvious nuclear pyknosis, nuclear fragmentation, and focal necrosis were observed, together with markedly reduced Ki‐67‐positive signals, indicating that CPAN effectively inhibited tumor tissue growth (Figure 5K and Figure S16a).

**FIGURE 5:**
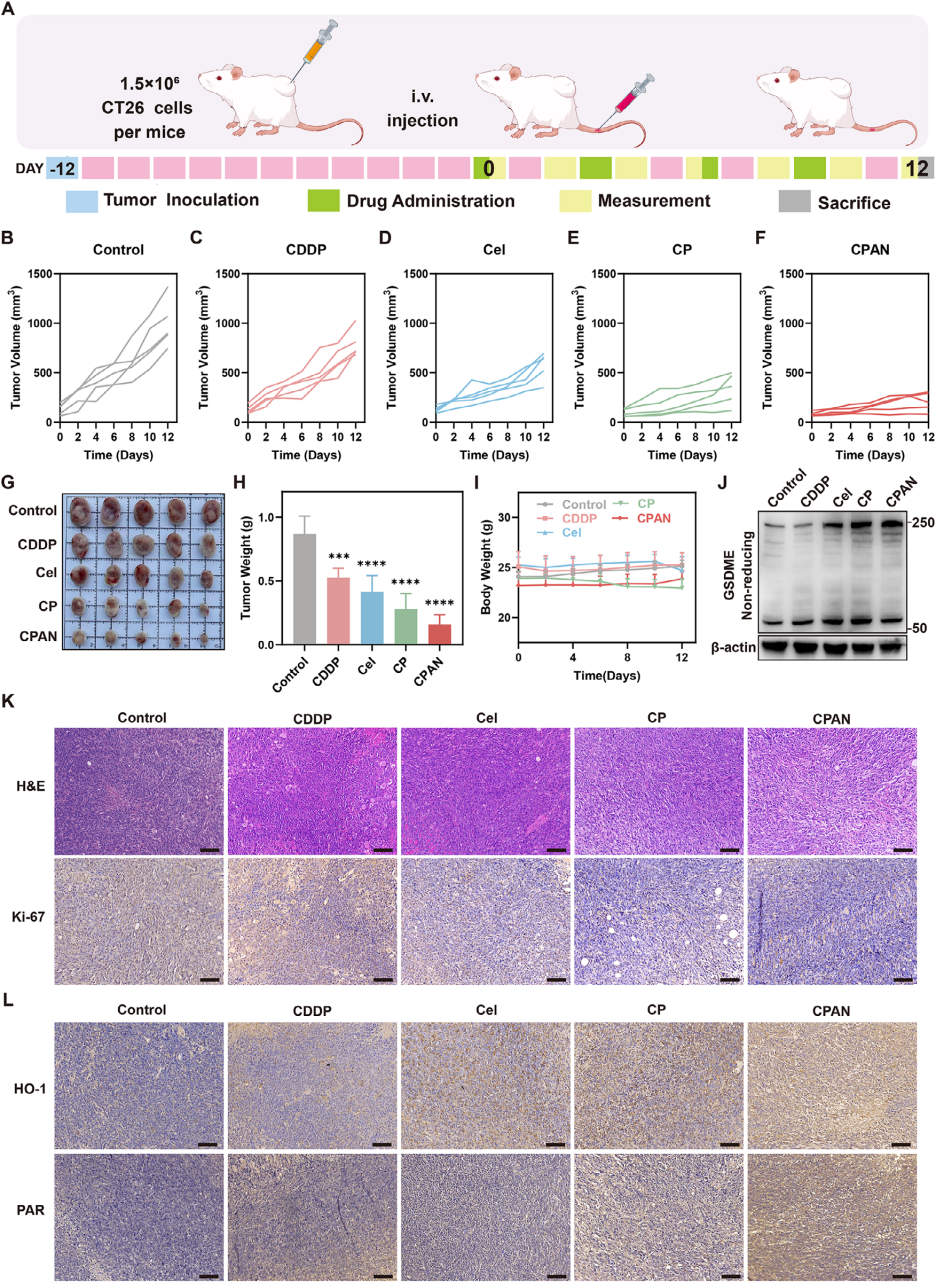
Antitumor performance of CPAN in CT26 tumor‐bearing mice. (A) Treatment scheme for CT26 subcutaneous tumor establishment, dosing, monitoring, and sample collection. The doses of Cel and CDDP were 3.0 and 2.0 mg kg^−^^1^, respectively. CP and CPAN were administered at equivalent Cel doses. (B–F) Tumor‐growth trajectories of individual animals receiving Control, CDDP, Cel, CP, or CPAN. (G) Excised tumors collected at the experimental endpoint. (H) Endpoint tumor weights. (I) Longitudinal changes in mouse body weight. (J) Non‐reducing immunoblot detection of GSDME oligomers in tumor tissue. (K) H&E staining and Ki‐67 immunohistochemistry of tumor sections (scale bar, 100 µm). (L) Immunohistochemical assessment of HO‐1 and PAR (scale bar, 100 µm). CP indicates the free combination of CDDP and Cel. Data are mean ± SD (*n* = 5 for H and I). Tumor weights were analyzed by one‐way ANOVA and body‐weight data by two‐way ANOVA. *p < 0.05, **p < 0.01, ***p < 0.001, ****p < 0.0001.

To determine whether CPAN induced ferroptosis and caspase‐independent pyroptosis in vivo in a manner consistent with the in vitro findings, total protein was extracted from tumor tissues from each group and analyzed by non‐reducing sodium dodecyl sulfate–polyacrylamide gel electrophoresis (SDS‐PAGE). High‐molecular‐weight GSDME oligomers were markedly increased in tumor tissues from the CPAN group compared with the Control, CDDP, Cel, and CP groups (Figure 5J and Figure S17). Immunohistochemical staining of tumor tissues further showed that HO‐1 and total PAR signals were markedly enhanced in the CPAN group (Figure 5L and Figure S16b,c). These results are consistent with the in vitro findings and suggest that CPAN also induces ferroptosis and caspase‐independent pyroptosis in vivo, contributing to its antitumor effect.

### 2.6 CPAN Remodels the Tumor Immune Microenvironment and Enhances Antitumor Immune Effects

After exploring the antitumor mechanism, we further investigated the immunomodulatory function of CPAN. The proportion of mature dendritic cells (CD80^+^CD86^+^) in tumors from CPAN‐treated mice was 1.9‐fold higher than in the Control group and was higher than in the other drug‐treated groups (Figure 6A,B). We also analyzed T‐cell infiltration in mouse tumor tissues. The proportion of CD3^+^CD8^+^ T cells in tumors from CPAN‐treated mice was 13.8%, which was 2.47‐, 1.39‐, and 1.25‐fold higher than in the Control, CDDP, and Cel groups, respectively (Figure 6C,D). At the same time, the Treg‐cell fraction decreased markedly in the CPAN group, from 8.29% in the Control group to 1.51% (Figure 6E,F). Thus, CPAN not only enhances effector CD8^+^ T‐cell infiltration but also weakens Treg‐mediated immunosuppression, thereby improving the tumor immune microenvironment.

**FIGURE 6:**
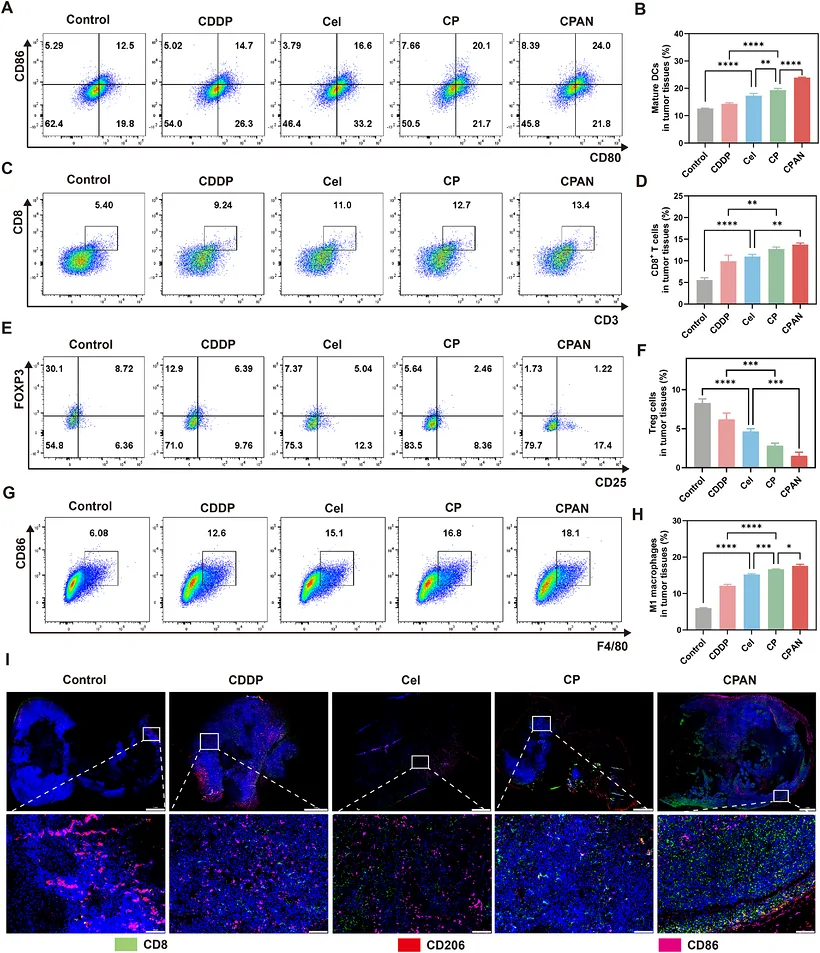
CPAN reshapes the CT26 tumor immune microenvironment and strengthens antitumor immunity. (A,B) Representative flow‐cytometry profiles and frequencies of CD80^+^CD86^+^ mature DCs. (C,D) Analysis of tumor‐infiltrating CD3^+^CD8^+^ T cells. (E,F) Frequencies of CD25^+^FOXP3^+^ Treg cells. (G,H) Quantification of F4/80^+^CD86^+^ M1 macrophages. (I) Multiplex immunofluorescence showing the distribution of CD8, CD86, and CD206‐associated signals within tumor tissues (scale bars, 1 mm, upper images; 100 µm, lower images). CP refers to free CDDP plus Cel. Values are presented as mean ± SD (*n* = 3), with group comparisons performed by one‐way ANOVA. *p < 0.05, **p < 0.01, ***p < 0.001, ****p < 0.0001.

Tumor‐associated macrophages (TAMs) are important immune‐cell populations in the tumor immune microenvironment, and their phenotypic state is closely related to tumor progression and antitumor immune responses [38]. Activated macrophages mainly include M1 and M2 phenotypes. M1 macrophages can phagocytose cancer cells and recruit T cells, whereas M2 macrophages promote tumor growth, invasion, and metastasis [39]. Promoting TAM polarization toward the M1 phenotype is therefore considered an important strategy for improving the tumor immune microenvironment. ROS production has been reported to induce macrophage polarization toward the M1 phenotype and effectively reverse an immunosuppressive microenvironment [40, 41]. Because our preceding results showed that CPAN promotes cellular ROS generation and accumulation, we evaluated the proportion of M1 macrophages in tumor tissues. CPAN treatment significantly increased the M1 macrophage fraction compared with the other treatment groups (Figure 6G). The proportion of M1 macrophages in tumor tissues from CPAN‐treated mice was threefold that of the Control group (Figure 6H). This change suggests that CPAN may promote M1 polarization of TAMs through ROS generation and thereby induce antitumor immune responses. Finally, multiplex immunofluorescence staining further validated this immune remodeling. In the Control group, CD8 and CD86 signals in tumor tissues were weak, whereas CD206 signals were relatively strong, indicating a strongly immunosuppressive tumor microenvironment. After CPAN treatment, CD8 and CD86 signals increased compared with the other treatment groups, whereas CD206 signals decreased (Figure 6I and Figure S18). Overall, these results indicate that CPAN effectively remodels the tumor immune microenvironment and enhances antitumor immune effects.

### 2.7 CPAN Enhances the Efficacy of Anti‐PD‐1 Immunotherapy

The preceding results showed that CPAN promoted DC maturation, increased CD8^+^ T‐cell infiltration, and alleviated immunosuppression in the tumor microenvironment. We therefore investigated whether these immunomodulatory effects could further enhance the therapeutic efficacy of anti‐PD‐1 antibody (aPD‐1) in CT26 tumor‐bearing mice. CT26 is commonly used as a pMMR/MSS syngeneic CRC model and has been reported to exhibit variable but measurable responsiveness to PD‐1/PD‐L1 blockade in different experimental settings [42, 43, 44]. CT26 tumor‐bearing mice were randomly assigned to Control, anti‐PD‐1, CPAN, and CPAN + anti‐PD‐1 (Combo) groups and treated according to the scheduled regimen (Figure 7A). As shown in Figure 7B,C, anti‐PD‐1 monotherapy delayed tumor growth, while CPAN produced a stronger inhibitory effect. Notably, tumor growth was further suppressed in the combination group. Endpoint tumor‐weight analysis showed that tumor weights decreased to 0.32 g in the anti‐PD‐1 group and 0.11 g in the CPAN group, and were further reduced to 0.04 g in the combination group (Figure 7D). No obvious body‐weight loss was observed in any group during treatment, suggesting good in vivo tolerability of the combination regimen (Figure 7E).

**FIGURE 7:**
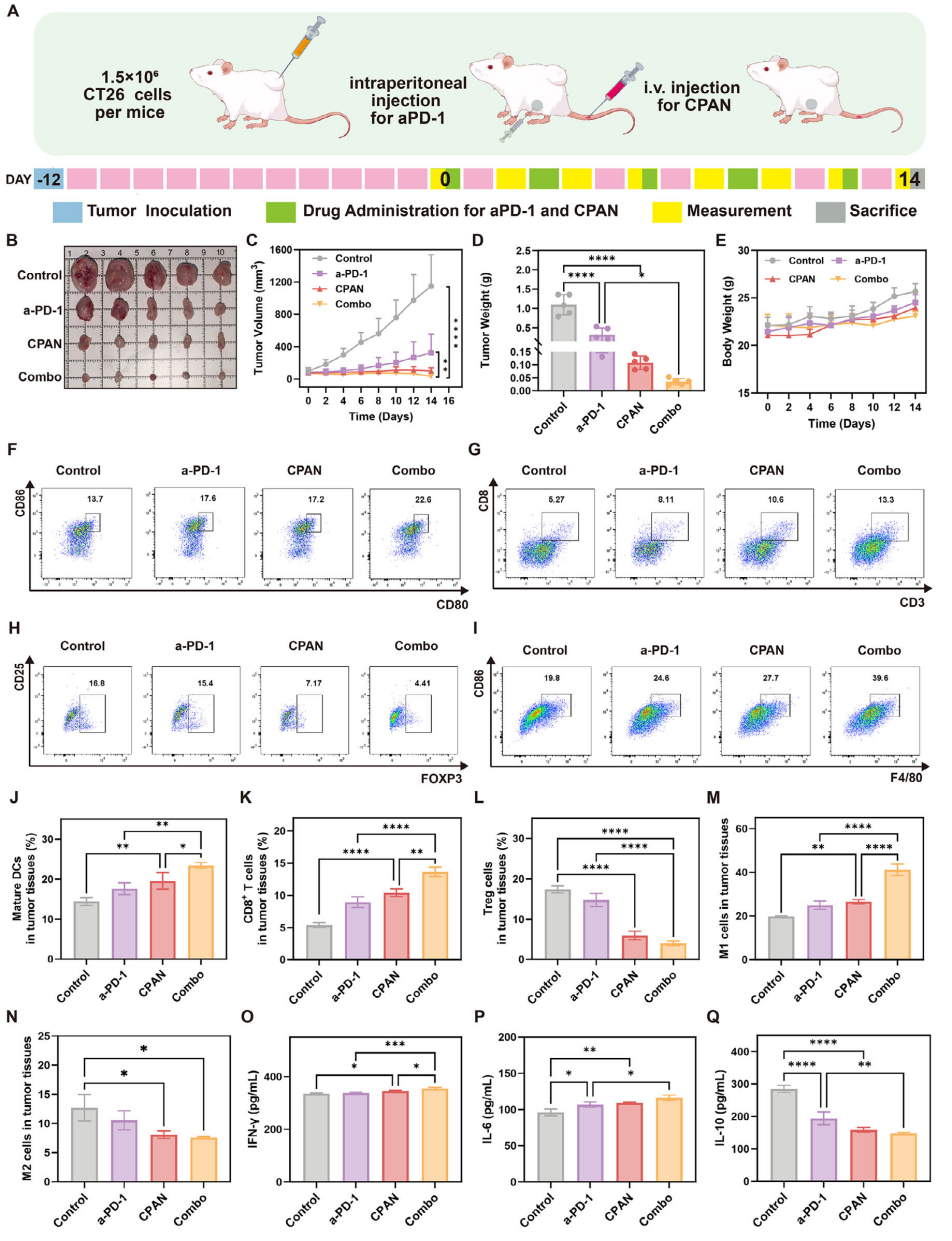
CPAN enhances the antitumor efficacy of anti‐PD‐1 immunotherapy. (A) Treatment and sampling schedule for CT26‐bearing mice receiving CPAN and/or anti‐PD‐1 (aPD‐1). (B) Representative excised tumors at the experimental endpoint. (C) Tumor‐growth curves. (D) Final tumor weights. (E) Body‐weight trajectories during treatment. (F,J) Representative flow‐cytometry profiles and quantification of CD80^+^CD86^+^ mature DCs. (G,K) Frequencies of CD3^+^CD8^+^ T cells. (H,L) Frequencies of CD25^+^FOXP3^+^ Treg cells. (I,M,N) Quantification of F4/80^+^CD86^+^ M1 and F4/80^+^CD206^+^ M2 macrophages. (O–Q) Serum concentrations of IFN‐γ, IL‐6, and IL‐10 at the endpoint. Combo denotes CPAN plus anti‐PD‐1. Values are mean ± SD (*n* = 5 for C–E; *n* = 3 for J–Q). Tumor‐growth data were analyzed by two‐way ANOVA, whereas endpoint comparisons in (D–Q) used one‐way ANOVA. *p < 0.05, **p < 0.01, ***p < 0.001, ****p < 0.0001.

To explore the potential mechanism by which CPAN sensitized tumors to anti‐PD‐1 immunotherapy, we analyzed immune‐cell populations in tumor tissues by flow cytometry. The proportion of CD80^+^CD86^+^ mature DCs increased to 23.4% in the combination group, exceeding that in the Control group (14.4%) and the monotherapy groups (Figure 7F,J). This result suggests that CPAN combined with anti‐PD‐1 further enhances local antigen presentation in tumors. At the T‐cell response level, combination treatment significantly increased the proportion of CD8^+^ T cells from 5.4% in the Control group to 13.7% (Figure 7G,K), while reducing the Treg‐cell fraction from 17.4% to 3.97% (Figure 7H,L). These findings indicate that combination treatment both enhances effector T‐cell infiltration and weakens Treg‐mediated immunosuppression. Macrophage phenotype analysis further showed that, compared with the other treatment groups, the combination group had a marked increase in M1 macrophages and a decrease in M2 macrophages (Figure 7I,M,N and Figure S19a). The M1/M2 macrophage ratio in tumor tissues from mice receiving the combination treatment was 3.6‐fold that of the Control group (Figure S19b).

In addition, serum samples were collected from mice, and levels of immune‐related cytokines, including interferon‐γ (IFN‐γ), interleukin‐6 (IL‐6), and interleukin‐10 (IL‐10), were measured. High IFN‐γ levels can inhibit tumor‐cell growth, stimulate cytotoxic T‐lymphocyte proliferation, and enhance systemic antitumor immunotherapy [45]. CPAN increased IFN‐γ levels, and this effect was more pronounced when CPAN was combined with anti‐PD‐1 (Figure 7O). Meanwhile, IL‐6 levels increased and IL‐10 levels decreased after CPAN and combination treatment (Figure 7P,Q). These results indicate that CPAN effectively remodels the tumor immune microenvironment and significantly enhances the antitumor efficacy of PD‐1 blockade in the CT26 syngeneic tumor model. By enhancing antitumor immune responses and alleviating immunosuppression, CPAN may provide a strategy for improving CRC immunotherapy.

## 3 Conclusion

In this study, we developed a hyaluronic acid‐modified celastrol–platinum(IV) self‐assembled nanoprodrug (CPAN) to enhance colorectal cancer immunotherapy. CPAN coordinated celastrol‐driven oxidative stress and lipid peroxidation with Pt(IV)‐associated DNA damage and PARP/PARylation signaling. This combination promoted ferroptosis and caspase‐independent pyroptotic death associated with full‐length GSDME oligomerization. The coupled cell‐death program enhanced immunogenic cell death, dendritic cell maturation, and antitumor T‐cell responses. In mice bearing CT26 syngeneic tumors, CPAN increased CD8^+^ T‐cell infiltration and the M1 macrophage fraction while reducing the Treg fraction. CPAN also increased serum IFN‐γ levels and decreased serum IL‐10 levels. These findings indicate remodeling of the tumor immune microenvironment, accompanied by enhanced therapeutic efficacy of PD‐1 blockade in this model. Collectively, these findings establish a mechanistically integrated nanoprodrug strategy linking ferroptotic lipid peroxidation to caspase‐independent, GSDME‐associated pyroptotic death to enhance tumor immunogenicity.

However, further validation in additional immunotherapy‐resistant and orthotopic CRC models is needed to assess the broader applicability and translational potential of this strategy. Because CD8^+^ T‐cell depletion was not performed, further experiments are needed to determine whether the combination's antitumor effect strictly depends on these cells.

## 4 Experimental Section

Experimental details are included in Supporting Information.

## Author Contributions

**Huirong Li**: conceptualization, methodology, investigation, validation, formal analysis, visualization, Writing – original draft, Writing – review and editing. **Wenxiang Feng**: investigation, validation, writing – original draft. **Le Xin**: data curation, visualization, investigation, writing – original draft. **Wu Zhong**: formal analysis, visualization, investigation, writing – original draft. **Zhuhuai Peng**: investigation. **Zhihong Ke**: validation. **Wenbin Zhou**: visualization. **Yutong Wu**: validation. **Ying Chen**: methodology. **Hongwei Wang**: data curation. **Jidong Liu**: formal analysis. **Jinjun Ye**: funding acquisition, resources, supervision. **Weihong Guo**: writing – review and editing, conceptualization, supervision, project administration.

## Conflicts of Interest

The authors declare no conflicts of interest.

## Supporting information




**Supporting File**: advs78681‐sup‐0001‐SuppMat.docx

## Data Availability

The RNA‐seq data generated in this study have been deposited in the NCBI Gene Expression Omnibus (GEO) database under accession number GSE350470. Other data that support the findings of this study are available from the corresponding author upon reasonable request.

## References

[1] F. Bray , M. Laversanne , H. Sung , et al., “Global Cancer Statistics 2022: GLOBOCAN Estimates of Incidence and Mortality Worldwide for 36 Cancers in 185 Countries,” CA: A Cancer Journal for Clinicians 74, no. 3 (2024): 229–263, 10.3322/caac.21834. 38572751

[2] M. Chalabi , Y. L. Verschoor , P. B. Tan , et al., “Neoadjuvant Immunotherapy in Locally Advanced Mismatch Repair–Deficient Colon Cancer,” New England Journal of Medicine 390, no. 21 (2024): 1949–1958, 10.1056/nejmoa2400634. 38838311

[3] D. T. Le , J. N. Uram , H. Wang , et al., “PD‐1 Blockade in Tumors with Mismatch‐Repair Deficiency,” New England Journal of Medicine 372, no. 26 (2015): 2509–2520, 10.1056/nejmoa1500596. PMC4481136 26028255

[4] T. André , K.‐K. Shiu , T. W. Kim , et al., “Pembrolizumab in Microsatellite‐Instability–High Advanced Colorectal Cancer,” New England Journal of Medicine 383, no. 23 (2020): 2207–2218, 10.1056/NEJMoa2017699. 33264544

[5] X. Wang , Y. Fang , W. Liang , et al., “Fusobacterium Nucleatum Facilitates Anti‐PD‐1 Therapy in Microsatellite Stable Colorectal Cancer,” Cancer Cell 42, no. 10 (2024): 1729–1746.e8, 10.1016/j.ccell.2024.08.019. 39303724

[6] Y. Chen , H. Jia , X. Zhang , et al., “Disruption of GPSM1/CSF1 Signaling Reprograms Tumor‐Associated Macrophages to Overcome Anti‐PD‐1 Resistance in Colorectal Cancer,” Journal for ImmunoTherapy of Cancer 13, no. 2 (2025): 010826, 10.1136/jitc-2024-010826. PMC12083360 40010765

[7] E. Chen and W. Zhou , “Immunotherapy in Microsatellite‐Stable Colorectal Cancer: Strategies to Overcome Resistance,” Critical Reviews in Oncology/Hematology 212 (2025): 104775, 10.1016/j.critrevonc.2025.104775. 40409481

[8] Q.‐L. Wang , Z. Chen , X. Lu , et al., “Methionine Metabolism Dictates PCSK9 Expression and Antitumor Potency of PD‐1 Blockade in MSS Colorectal Cancer,” Advanced Science 12, no. 19 (2025): 2501623, 10.1002/advs.202501623. PMC12097065 40125618

[9] A. Esposito , A. Agostini , G. Quero , et al., “Colorectal Cancer Patients‐Derived Immunity‐Organoid Platform Unveils Cancer‐Specific Tissue Markers Associated with Immunotherapy Resistance,” Cell Death & Disease 15, no. 12 (2024): 878, 10.1038/s41419-024-07266-5. PMC11618451 39632825

[10] J.‐D. Fumet , C. Latour , L. Nuttin , et al., “Tumor‐Associated Macrophages Produce PGE2 to Promote CD8+ T‐Cell Exhaustion and Drive Resistance to PD‐L1 Blockade in Microsatellite‐Stable Colorectal Cancer,” Cancer Research 86, no. 3 (2026): 785–801, 10.1158/0008-5472.can-25-0079. 41196020

[11] T. Sha , Y. Tang , X. Hu , H. Zhou , and L. Rao , “Regulating Gut Microbiota with Nanomaterials for Cancer Immunotherapy,” Interdisciplinary Medicine 3, no. 2 (2025): 20240103, 10.1002/inmd.20240103.

[12] S. J. Dixon , K. M. Lemberg , M. R. Lamprecht , et al., “Ferroptosis: An Iron‐Dependent Form of Nonapoptotic Cell Death,” Cell 149, no. 5 (2012): 1060–1072, 10.1016/j.cell.2012.03.042. PMC3367386 22632970

[13] G. Lei , L. Zhuang , and B. Gan , “Targeting Ferroptosis as a Vulnerability in Cancer,” Nature Reviews Cancer 22, no. 7 (2022): 381–396, 10.1038/s41568-022-00459-0. PMC10243716 35338310

[14] C. Conche , F. Finkelmeier , M. Pesic , et al., “Combining Ferroptosis Induction with MDSC Blockade Renders Primary Tumours and Metastases in Liver Sensitive to Immune Checkpoint Blockade,” Gut 72, no. 9 (2023): 1774–1782, 10.1136/gutjnl-2022-327909. PMC10423492 36707233

[15] J. Xiao , N. Feng , Q. Li , et al., “Mitochondria‐Specific GPX4 Inhibition Enhances Ferroptosis and Antitumor Immunity,” Journal of Controlled Release 383 (2025): 113841, 10.1016/j.jconrel.2025.113841. 40373937

[16] Q. Ding , W. Tang , X. Li , et al., “Mitochondrial‐Targeted Brequinar Liposome Boosted Mitochondrial‐Related Ferroptosis for Promoting Checkpoint Blockade Immunotherapy in Bladder Cancer,” Journal of Controlled Release 363 (2023): 221–234, 10.1016/j.jconrel.2023.09.024. 37717657

[17] Y. Liu , D. Tao , M. Li , and Z. Luo , “Biomaterial‐Mediated Metabolic Regulation of Ferroptosis for Cancer Immunotherapy,” WIREs Nanomedicine and Nanobiotechnology 16, no. 6 (2024): 2010, 10.1002/wnan.2010. 39492611

[18] B. T. Cookson and M. A. Brennan , “Pro‐Inflammatory Programmed Cell Death,” Trends in Microbiology 9, no. 3 (2001): 113–114, 10.1016/s0966-842x(00)01936-3. 11303500

[19] Y. Wang , W. Gao , X. Shi , et al., “Chemotherapy Drugs Induce Pyroptosis through Caspase‐3 Cleavage of a Gasdermin,” Nature 547, no. 7661 (2017): 99–103, 10.1038/nature22393. 28459430

[20] X. Sun , X. Cai , S. Li , et al., “High‐Dose Ascorbic Acid Selectively Induces Pyroptosis in LKB1‐Deficient Lung Cancer and Sensitizes Immunotherapy,” Cell Reports Medicine 6, no. 9 (2025): 102291, 10.1016/j.xcrm.2025.102291. PMC12490232 40818456

[21] Y. Tu , H. Wu , C. Zhong , et al., “Pharmacological Activation of STAT1‐GSDME Pyroptotic Circuitry Reinforces Epigenetic Immunotherapy for Hepatocellular Carcinoma,” Gut 74, no. 4 (2025): 613–627, 10.1136/gutjnl-2024-332281. PMC12013592 39486886

[22] J. Shi , Y. Zhao , K. Wang , et al., “Cleavage of GSDMD by Inflammatory Caspases Determines Pyroptotic Cell Death,” Nature 526, no. 7575 (2015): 660–665, 10.1038/nature15514. 26375003

[23] Y. Hu , Y. Liu , L. Zong , et al., “The Multifaceted Roles of GSDME‐Mediated Pyroptosis in Cancer: Therapeutic Strategies and Persisting Obstacles,” Cell Death & Disease 14, no. 12 (2023): 836, 10.1038/s41419-023-06382-y. PMC10725489 38104141

[24] L. Flanagan , M. Meyer , J. Fay , et al., “Low Levels of Caspase‐3 Predict Favourable Response to 5FU‐Based Chemotherapy in Advanced Colorectal Cancer: Caspase‐3 Inhibition as a Therapeutic Approach,” Cell Death & Disease 7, no. 2 (2016): e2087–e2087, 10.1038/cddis.2016.7. PMC4849164 26844701

[25] M. Ehrenschwender , S. Bittner , K. Seibold , and H. Wajant , “XIAP‐Targeting Drugs Re‐Sensitize PIK3CA‐Mutated Colorectal Cancer Cells for Death Receptor‐Induced Apoptosis,” Cell Death & Disease 5, no. 12 (2014): e1570–e1570, 10.1038/cddis.2014.534. PMC4649844 25501831

[26] B. Zhou , Z.‐H. Jiang , M.‐R. Dai , et al., “Full‐Length GSDME Mediates Pyroptosis Independent From Cleavage,” Nature Cell Biology 26, no. 9 (2024): 1545–1557, 10.1038/s41556-024-01463-2. 38997456

[27] H. Xu , H. Zhao , C. Ding , et al., “Celastrol Suppresses Colorectal Cancer via Covalent Targeting Peroxiredoxin 1,” Signal Transduction and Targeted Therapy 8, no. 1 (2023): 51, 10.1038/s41392-022-01231-4. PMC9895061 36732502

[28] S. Lu , Y. Li , and Y. Yu , “Glutathione‐Scavenging Celastrol‐Cu Nanoparticles Induce Self‐Amplified Cuproptosis for Augmented Cancer Immunotherapy,” Advanced Materials 36, no. 35 (2024): 2404971, 10.1002/adma.202404971. 38935977

[29] H. Huang , L. Zhu , B. R. Reid , G. P. Drobny , and P. B. Hopkins , “Solution Structure of a Cisplatin‐Induced DNA Interstrand Cross‐Link,” Science 270, no. 5243 (1995): 1842–1845, 10.1126/science.270.5243.1842. 8525382

[30] X. Wen , R. Zhang , Y. Hu , et al., “Controlled Sequential in Situ Self‐Assembly and Disassembly of a Fluorogenic Cisplatin Prodrug for Cancer Theranostics,” Nature Communications 14, no. 1 (2023): 800, 10.1038/s41467-023-36469-1. PMC9925730 36781887

[31] X. Dong , W. Zhao , K. Sun , Y. Yuan , Y. Sun , and D. Wei , “Self‐Assembly of Peptide‐Lipid Platinum(IV) Prodrug Amphiphile for αvβ3 Integrin‐Targeting Chemotherapy,” Advanced Healthcare Materials 15, no. 25 (2026): 71306, 10.1002/adhm.71306. 42216428

[32] P. Luo , D. Liu , Q. Zhang , et al., “Celastrol Induces Ferroptosis in Activated HSCs to Ameliorate Hepatic Fibrosis via Targeting Peroxiredoxins and HO‐1,” Acta Pharmaceutica Sinica B 12, no. 5 (2022): 2300–2314, 10.1016/j.apsb.2021.12.007. PMC9136576 35646542

[33] W. Yin , H. Xu , Z. Bai , et al., “Inhibited Peroxidase Activity of Peroxiredoxin 1 by Palmitic Acid Exacerbates Nonalcoholic Steatohepatitis in Male Mice,” Nature Communications 16, no. 1 (2025): 598, 10.1038/s41467-025-55939-2. PMC11724923 39799115

[34] A. V. Menon , J. Liu , H. P. Tsai , et al., “Excess Heme Upregulates Heme Oxygenase 1 and Promotes Cardiac Ferroptosis in Mice with Sickle Cell Disease,” Blood 139, no. 6 (2022): 936–941, 10.1182/blood.2020008455. PMC8832481 34388243

[35] W. S. Yang , R. SriRamaratnam , M. E. Welsch , et al., “Regulation of Ferroptotic Cancer Cell Death by GPX4,” Cell 156, no. 1–2 (2014): 317–331, 10.1016/j.cell.2013.12.010. PMC4076414 24439385

[36] E. Catanzaro , M. Beltrán‐Visiedo , L. Galluzzi , and D. V. Krysko , “Immunogenicity of Cell Death and Cancer Immunotherapy with Immune Checkpoint Inhibitors,” Cellular & Molecular Immunology 22, no. 1 (2025): 24–39, 10.1038/s41423-024-01245-8. PMC11685666 39653769

[37] D. V. Krysko , A. D. Garg , A. Kaczmarek , O. Krysko , P. Agostinis , and P. Vandenabeele , “Immunogenic Cell Death and DAMPs in Cancer Therapy,” Nature Reviews Cancer 12, no. 12 (2012): 860–875, 10.1038/nrc3380. 23151605

[38] R. M. L. Coelho , R. Debets , and D. Hammerl , “Tumor‐Associated Macrophages: Untapped Molecular Targets to Improve T Cell‐Based Immunotherapy,” Molecular Cancer 24, no. 1 (2025): 261, 10.1186/s12943-025-02481-w. PMC12532839 41102756

[39] A. Mantovani , P. Allavena , F. Marchesi , and C. Garlanda , “Macrophages as Tools and Targets in Cancer Therapy,” Nature Reviews Drug Discovery 21, no. 11 (2022): 799–820, 10.1038/s41573-022-00520-5. PMC9380983 35974096

[40] C. Shi , T. Liu , Z. Guo , R. Zhuang , X. Zhang , and X. Chen , “Reprogramming Tumor‐Associated Macrophages by Nanoparticle‐Based Reactive Oxygen Species Photogeneration,” Nano Letters 18, no. 11 (2018): 7330–7342, 10.1021/acs.nanolett.8b03568. 30339753

[41] M. Wang , M. Chang , C. Li , et al., “Tumor‐Microenvironment‐Activated Reactive Oxygen Species Amplifier for Enzymatic Cascade Cancer Starvation/Chemodynamic /Immunotherapy,” Advanced Materials 34, no. 4 (2022): 2106010, 10.1002/adma.202106010. 34699627

[42] E. M. Thomas , J. A. Wright , S. J. Blake , A. J. Page , D. L. Worthley , and S. L. Woods , “Advancing Translational Research for Colorectal Immuno‐Oncology,” British Journal of Cancer 129, no. 9 (2023): 1442–1450, 10.1038/s41416-023-02392-x. PMC10628092 37563222

[43] Y. Sato , Y. Fu , H. Liu , M. Y. Lee , and M. H. Shaw , “Tumor‐Immune Profiling of CT‐26 and Colon 26 Syngeneic Mouse Models Reveals Mechanism of Anti‐PD‐1 Response,” BMC Cancer 21, no. 1 (2021): 1222, 10.1186/s12885-021-08974-3. PMC8590766 34774008

[44] C.‐M. Tu , M.‐H. Lin , C.‐P. Liu , et al., “Intravenous Administration of CpG7909 Lipoplex Enhances Anti‐PD1 Immunotherapy by Modulating the Tumor Microenvironment and Inducing Durable Tumor Regression,” Scientific Reports 15, no. 1 (2025): 45354, 10.1038/s41598-025-29622-x. PMC12749086 41291120

[45] Y. Tang , J. Wei , X. Ge , et al., “Intratumoral Injection of Interferon Gamma Promotes the Efficacy of Anti‐PD1 Treatment in Colorectal Cancer,” Cancer Letters 588 (2024): 216798, 10.1016/j.canlet.2024.216798. 38467181

