# Supplementary material for: A Celastrol–Platinum(IV) Nanoprodrug Couples Ferroptosis With Caspase‐Independent Pyroptosis to Enhance Anti‐PD‐1 Therapy in Colorectal Cancer
Source: Adv Sci (Weinh). 2026 Nov 9:e78681. Online ahead of print. doi: 10.1002/advs.78681 (PMC13748766; doi:10.1002/advs.78681)
Supplement: Supplementary file 1 [file ADVS-9999-e78681-s001.docx]

Supporting Information

**A Celastrol–Platinum(IV) Nanoprodrug Couples Ferroptosis with Caspase-Independent Pyroptosis to Enhance Anti-PD-1 Therapy in Colorectal Cancer**

Huirong Li^1#^, Wenxiang Feng^2#^, Le Xin^1#^, Wu Zhong^2#^, Zhuhuai Peng^1^, Zhihong Ke^2^, Wenbin Zhou^1^, Yutong Wu^2^, Ying Chen^2^, Hongwei Wang^1^, Jidong Liu^1^, Jinjun Ye^1^*, Weihong Guo^2^*

^1^ Department of General Surgery, Shenzhen Clinical College (Longgang Central Hospital of Shenzhen), Guangzhou University of Chinese Medicine, Shenzhen 518116, China

^2^ Department of Gastroenterological Surgery, Ganzhou Hospital-Nanfang Hospital, Southern Medical University(GanzhouPeople's Hospital), Ganzhou 341400, China.

^#^Huirong Li, Wenxiang Feng, Le Xin and Wu Zhong contributed equally to this work.

Corresponding authors:

Jinjun Ye, Email: yejj610@163.com

Weihong Guo, Email: gwh2823@smu.edu.cn

**Experimental Section:**

**Materials and Reagents :**

Cisplatin (CDDP), celastrol (Cel), hyaluronic acid (HA, 10,000 Da), N-hydroxysuccinimide (NHS), 1-ethyl-3-(3-dimethylaminopropyl)carbodiimide (EDCI), and dimethyl sulfoxide (DMSO) were purchased from Shanghai Macklin Biochemical Technology Co., Ltd. Dialysis tubing with a molecular weight cutoff of 3,500 Da was obtained from Beyotime Biotechnology (Shanghai, China). Beyotime Biotechnology also supplied the reactive oxygen species (ROS), adenosine triphosphate (ATP), glutathione (GSH), and lactate dehydrogenase (LDH) assay kits, the Calcein-AM/propidium iodide (PI) live/dead staining kit, and BODIPY 581/591 C11. The hydrogen peroxide assay kit and 4′,6-diamidino-2-phenylindole (DAPI) staining solution were obtained from Beijing Solarbio Science & Technology Co., Ltd. XAV-939, ZnPPIX, Liproxstatin-1, Z-VAD-FMK, and recombinant mouse PRDX1 (Cat. No. HY-P73713) were purchased from MedChemExpress (MCE, USA).

Primary and secondary antibodies used in this study were obtained from the manufacturers listed in **Table S1**. The Rabbit IgG Nanoselector® Magnetic Beads IP Kit (Cat. No. 025-101-003K), including Rabbit IgG Nanoselector® magnetic beads, rabbit IgG isotype control, and an IP-compatible VHH-HRP secondary antibody, was purchased from AlpVHHs Bioscience Technology Co., Ltd. and used for co-immunoprecipitation assays.

**Table S1.** Antibodies used in this study.

| Target | Application | Manufacturer | Catalogue | Clone |
| --- | --- | --- | --- | --- |
| GSDME | WB | Proteintech | 67731-1-Ig | No. 1D9C3 |
| GSDME | Co-IP | Proteintech | 13075-1-AP | Rabbit polyclonal |
| GPX4 | WB/IHC | Proteintech | 30388-1-AP | Rabbit polyclonal |
| HO-1 | WB/IHC | Proteintech | 10701-1-AP | Rabbit polyclonal |
| PAR | WB/Co-IP/IHC | R&D Systems | 4335-MC-100 | 10HA |
| PARP1 | WB | Proteintech | 13371-1-AP | Rabbit polyclonal |
| CD44 | WB/IF | ChangYi biological | CYRM0279 | CYKL0279 |
| β-actin | WB | Proteintech | 66009-1-Ig | No. 2D4H5 |
| CD45 | Flow cytometry | BD Biosciences | 557659 | 30-F11 |
| CD3 | Flow cytometry | BD Biosciences | 553066 | 145-2C11 |
| CD4 | Flow cytometry | BD Biosciences | 557307 | GK1.5 |
| CD8 | Flow cytometry | BD Biosciences | 551162 | 53-6.7 |
| CD25 | Flow cytometry | BD Biosciences | 564571 | 7D4 |
| Foxp3 | Flow cytometry | BD Biosciences | 563101 | R16-715 |
| CD11c | Flow cytometry | BD Biosciences | 558079 | HL3 |
| CD11b | Flow cytometry | BD Biosciences | 550993 | M1/70 |
| CD80 | Flow cytometry | BD Biosciences | 553769 | 16-10A1 |
| CD86 | Flow cytometry | BD Biosciences | 558703 | GL1 |
| CD86 | Flow cytometry | BD Biosciences | 561962 | GL1 |
| F4/80 | Flow cytometry | BD Biosciences | 565410 | T45-2342 |
| CD206 | Flow cytometry | BD Biosciences | 565250 | MR5D3 |
| CD8 | IF | Proteintech | 29896-1-AP | Rabbit polyclonal |
| CD86 | IF | Proteintech | 30691-1-AP | Rabbit polyclonal |
| CD206 | IF | Proteintech | 18704-1-AP | Rabbit polyclonal |
| Ki-67 | IHC | Abcam | ab15580 | Rabbit polyclonal |
| HMGB1 | IF | Cell Signaling Technology | 3935S | Rabbit polyclonal |
| CRT | IF | Cell Signaling Technology | 12238S | Rabbit polyclonal |
| Goat anti-rabbit IgG-HRP | WB | Proteintech | SA00001-2 | — |
| Goat anti-mouse IgG-HRP | WB | Proteintech | SA00001-1 | — |
| Goat Anti-Rabbit/Mouse Universal Recombinant Secondary Antibody | IHC | Proteintech | RGAU011 | — |
| Goat anti-rabbit IgG-FITC | IF | Affinity | S0008 | — |
| Anti-Mouse PD-1 Antibody | in vivo | MCE | HY-P99144 | — |

**Cell Culture and Treatment :** CT26 murine colon cancer cells from the American Type Culture Collection (ATCC) were maintained in Dulbecco's modified Eagle's medium (DMEM) containing 10% fetal bovine serum (FBS), 100 IU/mL penicillin, and 100 μg/mL streptomycin. Cells were cultured at 37 °C in a humidified 5% CO₂ atmosphere. At approximately 80% confluence, cells were detached with trypsin and replated in flasks or experimental plates. For comparisons among CDDP, Cel, CP, and CPAN, dosing was normalized to the celastrol equivalent, with CDDP:Cel maintained at 1:1. In the main text, CP refers to the physical combination of celastrol and cisplatin.

**Synthesis of DPH, CPH, and CPA :** Dihydroxyplatinum(IV) (DPH) was synthesized by oxidizing CDDP. CDDP (300 mg) was suspended in 16 mL deionized water, and 4 mL H₂O₂ (30% w/v) was added. The suspension was protected from light and stirred at 75 °C for 8 h. After the mixture returned to room temperature, it was kept at 4 °C overnight to promote crystallization. The resulting crystals were recovered by centrifugation, rinsed with acetone, and dried at low temperature to yield DPH. CPH was synthesized through esterification coupling of DPH with celastrol. Celastrol (63 mg, 0.14 mmol) was dissolved in 5 mL anhydrous N,N-dimethylformamide (DMF) under nitrogen and protected from light. EDCI·HCl (40.2 mg, 0.21 mmol, 1.5 equiv.) and NHS (19.3 mg, 0.168 mmol, 1.2 equiv.) were introduced, and the solution was stirred for 3-4 h at room temperature in the dark to activate the carboxyl group of celastrol. In parallel, DPH (46.8 mg, approximately 0.14 mmol) was dispersed in 2 mL anhydrous DMF, combined with 20 μL triethylamine (TEA), and sonicated at 50 °C for 20 min until a homogeneous suspension formed. This DPH suspension was added slowly to the activated celastrol solution, and the reaction was continued at 50 °C for 48 h in the dark. After cooling, residual DPH was removed by centrifugation. The supernatant was partially concentrated under reduced pressure, followed by addition of excess precooled deionized water to precipitate the product. The precipitate was collected, washed three times with deionized water, and vacuum freeze-dried to obtain CPH. CPA was generated by coupling CPH with HA. HA (25 mg) was dissolved in anhydrous formamide at 50 °C with stirring until the solution became clear. EDCI·HCl (18 mg) and NHS (11 mg) were then added, and the reaction was allowed to proceed for 2-4 h in the dark to activate the HA carboxyl groups. CPH (50 mg) and a small amount of TEA were subsequently added, and the mixture was maintained at 50 °C for 24 h under light protection. The completed reaction mixture was loaded into a dialysis bag (MWCO 3,500 Da) and dialyzed sequentially against ethanol/water mixtures and deionized water to remove small molecules. The purified dialysate was freeze-dried to afford the CPA prodrug.

**Preparation of CPAN :** CPAN nanoparticles were produced by nanoprecipitation-driven self-assembly of CPA in water. CPA was first dissolved in formamide at 2 mg/mL. The CPA solution (3 mL) was infused dropwise into 10-20 mL deionized water with a syringe pump at 0.2-0.5 mL/min while the aqueous phase was vigorously stirred at room temperature. Stirring was continued for 2 h after the addition finished to complete self-assembly. The dispersion was transferred to a dialysis bag (MWCO 3,500 Da) and dialyzed against deionized water for 24-48 h, removing formamide and unassembled small molecules and producing the final CPAN dispersion.

**Physicochemical Characterization of CPAN :** The morphology and elemental distribution of CPAN were characterized by transmission electron microscopy (TEM; JEM-2100F, JEOL, Japan) together with elemental mapping. Scanning electron microscopy (SEM; ZEISS Sigma 360, Germany) was additionally used to examine nanoparticle morphology. Hydrodynamic diameter was determined by dynamic light scattering (DLS). Composition and chemical structure were further evaluated using ultraviolet-visible (UV-Vis) spectroscopy (UV-2600, Shimadzu, Japan), Fourier-transform infrared (FTIR) spectroscopy (Nicolet iS5, Thermo Fisher Scientific), and proton nuclear magnetic resonance (1H NMR) spectroscopy (600 MHz, Bruker, Germany).

**Colloidal Stability, Storage Stability, and In Vitro Release:**

To evaluate the colloidal stability of CPAN under different dispersion conditions, freshly prepared CPAN was dispersed in deionized water, phosphate-buffered saline (PBS), or PBS supplemented with 5% bovine serum albumin (BSA) and incubated at 37 °C. The Z-average diameter and polydispersity index (PDI) were measured by dynamic light scattering on days 0, 1, 3, 5, and 7. To assess liquid-state storage stability, freshly prepared aqueous CPAN dispersions were stored at 4 or 25 °C for 7 d, and the Z-average diameter and PDI were measured on days 0, 1, 3, 5, and 7. Representative photographs were also recorded before and after storage to evaluate visible changes in dispersion appearance, including precipitation, aggregation, or phase separation. For solid-state stability, CPAN was lyophilized and stored at 25 °C under light-protected conditions. An aliquot of freshly lyophilized CPAN was immediately reconstituted in deionized water as the day-0 sample, while another aliquot was stored for 7 d before reconstitution. The Z-average diameter and PDI of the reconstituted samples were then measured by dynamic light scattering. All stability measurements were performed using three independent replicates.

Cel release from CPAN was tested by dialysis. CPAN (2 mg) was dispersed in 2 mL PBS containing 15% DMSO, placed in a dialysis bag (MWCO 3,500 Da), and immersed in 50 mL PBS containing 15% DMSO at pH 7.4, 6.5, or 5.3. The release system was stirred at 100 rpm at 37 °C in the dark. At 0, 1, 2, 4, 6, 8, 10, 16, and 24 h, 1 mL of external medium was removed and replaced with the same volume of fresh PBS/15% DMSO at the matched pH. The experiment was continued for 24 h. Cel concentration was quantified from absorbance at 424 nm using a UV-Vis spectrophotometer and a pre-established calibration curve. Cumulative release was calculated using the following equation:

$Cumulative release\left( \% \right)=\frac{1\times\sum_{i=1}^{n-1} C_{i}+50\times C_{n}}{weight of drug in nanosystems}\times100\%$	[1]

**Cellular Uptake Assay :** CT26 cells were plated in six-well plates or confocal dishes and allowed to attach overnight. Rhodamine B-labeled CPAN was added for 1, 4, or 7 h. Cells were then washed with PBS, and intracellular fluorescence was evaluated by confocal microscopy and flow cytometry. In the competitive uptake experiment, free HA was applied before fluorescent CPAN to occupy CD44 receptors, and fluorescence was measured after a 7 h incubation.

**Cell Viability and Live/Dead Staining :** For cytotoxicity testing, CT26 cells were plated in 96-well plates at 8,000 cells per well, cultured overnight, and exposed to serial concentrations of CDDP, Cel, or CPAN for 24 h. Cell Counting Kit-8 (CCK-8) reagent was then added for 1 h, and absorbance at 450 nm was recorded. Viability was expressed relative to untreated cells, and half-maximal inhibitory concentration (IC50) values were obtained by fitting a four-parameter nonlinear regression model. For live/dead imaging, treated cells were incubated with Calcein-AM and PI for 10 min according to the kit protocol, washed, and imaged immediately. Three randomly selected fields from each group were used to quantify live and dead cell proportions.

**Detection of GSH, ROS, Fe²⁺, and Lipid Peroxidation :** Cells were harvested after 24 h of treatment for analysis of GSH, ROS, Fe²⁺, and lipid peroxidation. GSH and Fe²⁺ were quantified with commercial assay kits and normalized to total protein. Intracellular ROS was assessed using the 2′,7′-dichlorodihydrofluorescein diacetate (DCFH-DA) probe; cells were incubated with 10 μmol/L DCFH-DA for 20 min, washed, imaged, and quantified by mean fluorescence intensity. Lipid peroxidation was monitored with BODIPY 581/591 C11. Cells were labeled with 2 μmol/L BODIPY 581/591 C11 for 20 min, washed, and imaged, after which mean green and red fluorescence intensities were measured and used to calculate the fluorescence ratio.

**LDH Release Assay :** CT26 cells were seeded into 96-well plates at 8,000 cells per well and cultured overnight at 37 °C under 5% CO₂. After attachment, cells received the indicated drug treatments for 24 h. Culture supernatant (100 μL) from each well was transferred to a fresh 96-well plate, mixed with an equal volume of LDH working reagent according to the lactate dehydrogenase (LDH) assay-kit protocol, and incubated for 30 min at 37 °C in the dark. Stop solution (20 μL) was added to end the reaction. Absorbance was measured at 450 nm with 600 nm as the reference wavelength on a microplate reader, and LDH concentration was then calculated.

**Recombinant PRDX1 Peroxide-Scavenging Assay :** Recombinant mouse PRDX1 (rmPRDX1) was used to determine whether celastrol affected PRDX1-dependent peroxide removal. rmPRDX1 was diluted in PBS (pH 7.4) and pretreated with celastrol, CDDP, or an equal volume of PBS at 37 °C for 30 min. H₂O₂ was added to start the reaction, and the final reaction volume was adjusted to 100 μL. After 5 min at room temperature, residual H₂O₂ was quantified using a commercial hydrogen peroxide assay kit. Activity measured in the PBS-treated rmPRDX1 group was set to 100%, and relative PRDX1 activity was calculated for each treatment.

**Western Blot Analysis :** For conventional Western blotting, CT26 cells and excised tumor tissues from each treatment group were processed on ice in radioimmunoprecipitation assay (RIPA) lysis buffer supplemented with protease and phosphatase inhibitors; tumor samples were homogenized in precooled lysis buffer. Lysates were centrifuged at 12,000 x g for 30 min at 4 °C, and the supernatants were collected for protein quantification using a bicinchoninic acid (BCA) assay. Protein samples (30 μg per lane) were resolved by sodium dodecyl sulfate-polyacrylamide gel electrophoresis (SDS-PAGE) and transferred onto polyvinylidene difluoride (PVDF) membranes. For CD44 expression validation, 15, 25, and 35 μg of total protein were loaded as indicated. Membranes were blocked for 1.5 h at room temperature in Tris-buffered saline with Tween 20 (TBST) containing 5% bovine serum albumin or 5% nonfat milk, followed by overnight incubation at 4 °C with primary antibodies against GPX4, HO-1, PARP1, poly(ADP-ribose), GSDME, CD44 or beta-actin. After TBST washes, HRP-conjugated secondary antibodies were applied for 1.5 h at room temperature. Signals were visualized with enhanced chemiluminescence (ECL) reagent and recorded using a chemiluminescence imaging system. ImageJ was used for densitometry, and target proteins were normalized to the corresponding loading controls. For GSDME oligomerization analysis, proteins were extracted from cells or tumor tissues using non-reducing lysis buffer containing protease inhibitors but no reducing agents. Equal protein amounts were mixed with non-reducing loading buffer lacking dithiothreitol (DTT) or beta-mercaptoethanol and were loaded without heat denaturation. Samples were separated by non-reducing SDS-PAGE, transferred to PVDF membranes, and detected with anti-GSDME antibody to visualize monomeric and high-molecular-weight oligomeric species.

**Co-immunoprecipitation assay :** Co-immunoprecipitation was performed using a Rabbit IgG Nanoselector® Magnetic Beads IP Kit according to the manufacturer’s instructions. Briefly, CT26 cells were treated with CDDP, Cel, CP, CPAN, or CPAN plus Veliparib for the indicated time. Both adherent and floating cells were collected and lysed in ice-cold lysis buffer supplemented with protease inhibitor cocktail and 1 mM PMSF. The cell lysates were incubated on ice for 30 min and centrifuged at 17,000 × g for 10 min at 4°C to remove insoluble debris. A portion of each lysate was retained as the input control. For immunoprecipitation, equal amounts of total protein from each group were incubated with anti-GSDME antibody at 4°C overnight. Rabbit IgG isotype control was used as the negative IP control. The antibody–protein complexes were then captured with pre-equilibrated Rabbit IgG Nanoselector® magnetic beads at 4°C for 1 h with gentle rotation. After magnetic separation, the beads were washed three times with wash buffer and transferred to a new tube during the final wash. The immunoprecipitated proteins were eluted by boiling the beads in 2× SDS sample buffer and then subjected to SDS-PAGE and Western blotting. For detection of GSDME-associated PARylation, the immunoprecipitated proteins were immunoblotted with an anti-poly(ADP-ribose) antibody. Immunoprecipitated GSDME was detected as the IP loading control. Total PAR, GSDME, and β-actin levels in whole-cell lysates were analyzed as input controls. An IP-compatible light-chain-specific VHH-HRP secondary antibody was used to minimize interference from the IgG heavy chain near 55 kDa.

**RNA Sequencing and Bioinformatic Analysis :** For RNA-seq, total RNA was isolated from CT26 cells exposed to PBS, CDDP, celastrol, or CPAN for 24 h, using three independent biological replicates per group. RNA quantity and OD260/280 values (1.7-2.2) were determined with a spectrophotometer. RNA integrity and possible genomic DNA contamination were checked by denaturing agarose gel electrophoresis. Samples meeting quality requirements underwent ribosomal RNA depletion before library preparation. Library integrity was verified on an Agilent 2100 Bioanalyzer. After quantification and quality control, 150 bp paired-end reads were generated on an Illumina NovaSeq 6000 platform. Normalized data were used to identify differentially expressed genes, followed by gene set enrichment analysis (GSEA), Gene Ontology (GO) enrichment, Kyoto Encyclopedia of Genes and Genomes (KEGG) pathway analysis, and protein-protein interaction (PPI) network construction.

**Immunofluorescence Assay :** CT26 cells were cultured in confocal dishes for 24 h at 37 °C. The medium was replaced with fresh medium containing PBS, CDDP, Cel, CP, or CPAN, and treatment was continued for another 24 h. Cells were washed with PBS and fixed in 4% paraformaldehyde for 30 min at room temperature. For CRT surface staining, permeabilization was omitted. For HMGB1 staining, fixed cells were permeabilized with 0.5% Triton X-100 for 20 min. For CD44 staining, untreated CT26 cells were processed in parallel under non-permeabilizing conditions to evaluate CD44 expression and membrane localization. After additional PBS washes, samples were blocked with 5% BSA for 1 h at room temperature and then incubated at 4 °C with primary antibodies against CRT, HMGB1 or CD44. Cells were washed again, incubated with the appropriate fluorescent secondary antibodies for 1.5 h at room temperature in the dark, counterstained with DAPI for 10 min in the dark, and imaged by confocal fluorescence microscopy.

**In Vitro Induction of Dendritic Cell Maturation :** Bone marrow cells were collected from femurs and tibias of 6- to 8-week-old C57 mice and seeded in six-well plates at 5 x 10⁵ cells per well. To generate bone marrow-derived dendritic cells (BMDCs), cells were cultured for 7 d at 37 °C in medium containing recombinant mouse granulocyte-macrophage colony-stimulating factor (rmGM-CSF; 20 ng/mL) and recombinant mouse interleukin-4 (rmIL-4; 10 ng/mL). CT26 cells were treated with PBS, CDDP, Cel, CP, or CPAN in complete medium. After 24 h, conditioned supernatants were collected and cocultured with prepared BMDCs for 48 h. BMDCs were harvested, labeled with fluorescent antibodies against CD80 and CD86 for 45 min at 4 °C, washed with PBS, resuspended, and examined by flow cytometry. Representative gating strategies used for flow-cytometric analysis are shown in Figure S20.

**Establishment and Treatment of the CT26 Subcutaneous Tumor Model :** Male BALB/c mice (6-8 weeks old) were used for CT26 subcutaneous tumor establishment. Log-phase CT26 cells were harvested, washed twice with sterile PBS, and resuspended in PBS. Each mouse received a subcutaneous injection of 1.5 x 10⁶ CT26 cells in 100 μL into the right dorsal flank. Tumor length and width were monitored with calipers, and volume was calculated as length x width²/2. When tumor volume approached 100 mm³, mice were randomized into Control, CDDP, Celastrol, CP, and CPAN groups or into Control, anti-PD-1 (aPD-1), CPAN, and CPAN + aPD-1 (Combo) groups, with five mice in each group. Treatments were delivered by tail-vein or intraperitoneal injection according to the planned schedules. Celastrol was administered at 3 mg/kg, and aPD-1 was administered at 10 mg/kg. Other treatment doses were adjusted to the corresponding celastrol equivalent, while the Control group received the same volume of PBS. Tumor volume and body weight were recorded every 2 d. At the predetermined endpoint, mice were euthanized; tumors were removed intact, photographed, and weighed; and tumor tissue and serum were collected for downstream analyses. No formal blinding was applied during treatment administration, tumor measurement, or subsequent data analysis. No animals or data points were excluded from the analysis. All animal procedures were approved by the Laboratory Animal Ethics Committee of Shenzhen Longgang Central Hospital (Approval No. 2025-122) and followed applicable animal-care and ethical guidelines.

**Biodistribution of CPAN :** Near-infrared fluorescence imaging was used to evaluate CPAN biodistribution and tumor targeting in vivo. Subcutaneous CT26 tumor-bearing male BALB/c mice (6 weeks old; tumor volume approximately 100 mm³) were randomized into two groups (n = 3) and intravenously injected with free Cy5.5 or Cy5.5-labeled CPAN (CPAN@Cy5.5). Fluorescence distribution was tracked with an in vivo imaging system (IVIS) at 2, 8, 16, and 24 h after injection. At 24 h, mice were euthanized, and the heart, liver, spleen, lung, kidney, and tumors were collected for ex vivo fluorescence imaging to compare nanoparticle accumulation among tissues.

**Hematoxylin and Eosin Staining :** Tumors and major organs (heart, liver, spleen, lung, and kidney) were excised from mice in each group and fixed in 4% paraformaldehyde for 48 h. Fixed samples were processed through graded ethanol dehydration, xylene clearing, paraffin embedding, and sectioning into serial 4-5 μm slices. Sections were deparaffinized with xylene, rehydrated through graded ethanol, stained sequentially with hematoxylin and eosin, dehydrated, cleared, and mounted in neutral resin. Images were obtained using a light microscope. Tumor sections were assessed for cellular morphology, tissue disruption, and necrosis, whereas major-organ sections were examined for treatment-related toxicity and pathological injury.

**Immunohistochemistry :** Paraffin-embedded tumor sections were deparaffinized, rehydrated, and subjected to heat-induced antigen retrieval in sodium citrate buffer. After cooling to room temperature, endogenous peroxidase activity was quenched with 3% H₂O₂, and sections were blocked with BSA blocking solution for 40 min at room temperature. Primary antibodies against Ki-67, HO-1, or poly(ADP-ribose) (PAR) were applied overnight at 4 °C. On the following day, sections were equilibrated to room temperature, washed with PBS, and incubated with the corresponding HRP-conjugated secondary antibodies. Signals were developed using 3,3′-diaminobenzidine, nuclei were counterstained with hematoxylin, and sections were dehydrated, cleared, mounted, and imaged under a light microscope.

**Flow-Cytometric Analysis of Tumor-Infiltrating Immune Cells :** Fresh tumor tissues were minced and digested for 30 min at 37 °C with shaking in digestion buffer containing collagenase IV and DNase I. The digest was passed through a 70 μm cell strainer, and cells were collected by centrifugation. Red blood cells were eliminated with red blood cell lysis buffer. The resulting single-cell suspensions were washed with PBS and stained with surface antibodies. Mature dendritic cells were identified with CD45, CD11c, CD80, and CD86; CD8⁺ T cells with CD45, CD3, and CD8; regulatory T cells with CD45, CD3, CD4, CD25, and Foxp3; and macrophage subsets with CD45, CD11b, F4/80, CD86, and CD206. For intracellular markers such as Foxp3, fixation, permeabilization, and intracellular staining were performed according to the fixation/permeabilization kit instructions after surface labeling. Stained cells were washed, resuspended in PBS, acquired by flow cytometry, and analyzed in FlowJo. Representative gating strategies used for flow-cytometric analysis are shown in Figure S20.

**Multiplex Immunofluorescence Staining :** Paraffin-embedded tumor sections were deparaffinized, rehydrated, and subjected to antigen retrieval, followed by blocking with blocking solution for 60 min at room temperature. Primary antibodies against CD8, CD86, and CD206 were then added sequentially, and corresponding secondary antibodies for different fluorescence channels were used for signal development. After each round of antibody staining, antibody stripping or inactivation was performed according to the instructions of the multiplex immunofluorescence kit before the next staining cycle. After all targets had been stained, nuclei were counterstained with DAPI, and sections were mounted with antifade mounting medium. Images were acquired using a multispectral fluorescence imaging system. At least three representative nonoverlapping fields were randomly selected from each section for analysis. CD8-, CD86-, and CD206-positive signals were quantified using the corresponding multiplex immunofluorescence analysis software.

**Statistical Analysis :** In vitro data are presented as mean ± standard deviation from three independent experiments. Mouse data are presented as mean ± standard deviation (SD) for five values where indicated. No statistical method was used to predetermine sample size, but sample sizes were similar to those reported in previous publications. In animal experiments, tumor-bearing mice were randomly assigned before treatment. No animals or data points were excluded from the analysis. All statistical analyses were performed using GraphPad Prism 10. Comparisons among multiple groups were performed using one-way analysis of variance (ANOVA) with Tukey's multiple-comparison test. Data involving two independent variables were analyzed by two-way ANOVA with Tukey's multiple-comparison test. *p* < 0.05 was considered statistically significant. Data distribution was assumed to be normal but was not formally tested. All Western blots shown were repeated at least twice.

**Figure S1.** Additional morphological characterization of CPAN. A) TEM image of CPAN. Scale bar: 500 nm. B) SEM image of CPAN. Scale bar: 200 nm.

**Figure S2.** Stability of CPAN under different dispersion and storage conditions. A,B) Changes in the Z-average diameter and polydispersity index (PDI) of CPAN dispersed in deionized water, PBS, or PBS containing 5% BSA during incubation at 37 °C for 7 d. C,D) Changes in the Z-average diameter and PDI of CPAN aqueous dispersions stored at 4 or 25 °C for 7 d. E,F) Z-average diameter and PDI of lyophilized CPAN after reconstitution before and after storage at 25 °C for 7 d. G) Representative photographs of freshly prepared CPAN dispersion and redispersed CPAN after lyophilization and storage at 4 or 25 °C for 7 d. Data are presented as mean ± SD (n = 3). ns, not significant.

**Figure S3.** CD44 expression in CT26 cells. A) Western blot analysis of CD44 expression in CT26 cells with different protein loading amounts (15, 25, and 35 μg). B) Representative immunofluorescence images of CD44 (green) and DAPI-stained nuclei (blue) in CT26 cells. Scale bar: 100 μm.

**Figure S4.** Cellular uptake of Rhodamine B-labeled CPAN in CT26 cells. Representative fluorescence images show time-dependent uptake after 1, 4, and 7 h of incubation and reduced uptake after pretreatment with free HA. Scale bar: 50 μm. Quantification shows the mean fluorescence intensity of rhodamine B. Data are presented as mean ± SD (n=3); *p* values were calculated by one-way ANOVA. ns, not significant; ***p* < 0.01.

**Figure S5.** Relative H₂O₂-scavenging activity of recombinant mouse PRDX1 after treatment with CDDP or celastrol. Data are presented as mean ± SD (n=3); ns, not significant; *p* values were calculated by one-way ANOVA. *****p* < 0.0001.

**Figure S6.** Intracellular ferrous ion (Fe²⁺) levels in CT26 cells after treatment with CDDP, Cel, CP, or CPAN. CP denotes the simple combination of CDDP and Cel. Data are presented as mean ± SD (n=3); *p* values were calculated by one-way ANOVA. *****p* < 0.0001.

**Figure S7.** Quantitative analysis of Western blot bands for ferroptosis- and pyroptosis-related proteins in CT26 cells after different treatments. a,b) Relative GPX4 and HO-1 expression. c,d) Relative PARP1 and PAR expression. e,f) Relative GSDME oligomer and monomer levels. Data are presented as mean ± SD (n=3); ns, not significant; *p* values were calculated by one-way ANOVA. **p* < 0.05; ***p* < 0.01; ****p* < 0.001; *****p* < 0.0001.

**Figure S8.** HO-1 inhibition attenuates CPAN-induced lipid peroxidation. Representative BODIPY 581/591 C11 fluorescence images and quantification of the oxidized/reduced fluorescence ratio in CT26 cells treated with CPAN with or without ZnPPIX. Scale bar: 200 μm Data are presented as mean ± SD (n=3); ns, not significant; *p* values were calculated by one-way ANOVA. *****p* < 0.0001.

**Figure S9.** Ferroptosis inhibition partially rescues CPAN-induced cytotoxicity. Cell viability of CT26 cells treated with CPAN with or without Liproxstatin-1 pretreatment was measured by CCK-8 assay. Data are presented as mean ± SD (n=3); ns, not significant; *p* values were calculated by one-way ANOVA. *****p* < 0.0001.

**Figure S10.** The PAR signal detected in GSDME immunoprecipitates was quantified and normalized to immunoprecipitated GSDME. Data are presented as mean ± SD (n=3); ns, not significant; *p* values were calculated by one-way ANOVA. *****p* < 0.0001.

**Figure S11.** LDH release from CT26 cells after the indicated treatments. Increased LDH release indicates plasma-membrane disruption associated with CPAN-induced cell death. Data are presented as mean ± SD (n=3); *p* values were calculated by one-way ANOVA. *****p* < 0.0001.

**Figure S12.** Bright-field microscopy images of CT26 cells after control or CPAN treatment. Red arrows indicate cell swelling-like morphological changes after CPAN treatment. Scale bar: 50μm.

**Figure S13.** Inhibitor effects on CPAN-induced GSDME oligomerization. Quantification of non-reducing Western blot bands showing relative GSDME oligomer and monomer levels after pretreatment with Veliparib, Liproxstatin-1 (Lip), or both. Data are presented as mean ± SD (n=3); ns, not significant; *p* values were calculated by one-way ANOVA. ***p* < 0.01; ****p* < 0.001.

**Figure S14.** In vivo and ex vivo fluorescence imaging of CPAN tumor accumulation. CT26 tumor-bearing mice were intravenously injected with free Cy5.5 or Cy5.5-labeled CPAN (CPAN@Cy5.5), and fluorescence distribution was monitored at 2, 8, 16, and 24 h. Major organs and tumors were imaged ex vivo at 24 h.

**Figure S15.** Biosafety evaluation of CPAN in mice. a) Serum biochemical indicators of liver and kidney function, including ALT, AST, CREA, and UREA. b) H&E staining of major organs from control and CPAN-treated mice. Scale bar:100μm. Data are presented as mean ± SD (n=3); *p* values were calculated by Student’s t-test. ns, not significant.

**Figure S16.** Quantification of tumor immunohistochemistry after in vivo treatment. Relative staining intensities of a) Ki-67, b) HO-1, and c) PAR in tumor tissues from Control, CDDP, Cel, CP, and CPAN groups. Data are presented as mean ± SD (n=3); ns, not significant; *p* values were calculated by one-way ANOVA. **p* < 0.05; ****p* < 0.001; *****p* < 0.0001.

**Figure S17.** Quantitative analysis of GSDME oligomerization in tumor tissues. Relative levels of a) GSDME oligomer and b) GSDME monomer were determined by non-reducing Western blotting after different in vivo treatments. Data are presented as mean ± SD (n=3); ns, not significant; *p* values were calculated by one-way ANOVA. ***p* < 0.01.

**Figure S18.** Quantitative analysis of multiplex immunofluorescence signals in tumor tissues. Percentages of CD8-, CD86-, and CD206-positive cells after treatment with Control, CDDP, Cel, CP, or CPAN are shown.

**Figure S19.** Macrophage remodeling after CPAN and anti-PD-1 combination therapy. a) Representative flow-cytometric plots of F4/80⁺CD206⁺ M2 macrophages in tumor tissues after Control, aPD-1, CPAN, or combination treatment. b) Quantification of the M1/M2 macrophage ratio. Data are presented as mean ± SD (n=3); *p* values were calculated by one-way ANOVA. ***p* < 0.01; *****p* < 0.0001.

**Figure S20.** Representative flow-cytometric gating strategies for BMDCs and tumor-infiltrating immune cells.
